# Supplementary material for: The geography of climate and the global patterns of species diversity
Source: Nature. 2023 Sep 27;622(7983):537–44. doi: 10.1038/s41586-023-06577-5 (PMC10584679; doi:10.1038/s41586-023-06577-5)
Supplement: Supplementary file 1 — Supplementary Methods (providing an expanded explanation of the methodology and additional sensitivity analyses, including in-depth discussions on the various definitions of climate space explored (Sensitivity analysis for various definitions of climate space), comparative analyses between linear and nonlinear models (Exploring nonlinear relationships), methodological intricacies in determining predictor importance (Measuring predictors importance) and Figs. 1–8 to augment our methodology and sensitivity analyses) and Tables 1–22 (showcasing comprehensive statistical results for all tetrapod groups across several climate space resolutions). [file 41586_2023_6577_MOESM1_ESM.pdf]

nature portfolio

https://doi.org/10.1038/s41586-023-06577-5

Supplementary information

The geography of climate and the global patterns of species diversity

In the format provided by the authors and unedited

Supplementary Information for

The geography of climate and the global patterns of species diversity

Marco Túlio P. Coelho, Elisa Barreto, Thiago F. Rangel, José Alexandre F. Diniz-Filho, Rafael O. Wüest, Wilhelmine Bach, Alexander Skeels, Ian R. McFadden, David W. Roberts, Loïc Pellissier, Niklaus E. Zimmermann, Catherine H. Graham

Marco Túlio Pacheco Coelho
(marcotpcoelho@gmail.com)

Description: This supplementary material provides an expanded explanation of our methodology and additional sensitivity analyses. It includes in-depth discussions on the various definitions of climate space explored (*Sensitivity Analysis for Various Definitions of Climate Space*, Page 1), comparative analyses between linear and non-linear models (*Exploring Non-Linear Relationships*, Page 1), methodological intricacies in determining predictor importance (*Measuring Predictors Importance*, Page 2), and *Supplementary Figures* (Page 4 to 12) to augment our methodology and sensitivity analyses. Moreover, it encompasses 22 *Supplementary Tables* (Pages 13 to 67) showcasing comprehensive statistical results for all tetrapod groups across multiple climate space resolutions.

Index

Supplementary Methods **1**
1. Sensitivity Analysis for Various Definitions of Climate Space 1
2. Exploring non-linear relationships 1
3. Measuring predictors importance 2
4. Supplementary Figures 4

Supplementary Tables **13**

Supplementary Methods

1. Sensitivity Analysis for Various Definitions of Climate Space

We conducted a set of sensitivity analyses based on different definitions of climate space. For each tetrapod group, we employed a Generalized Additive Model (GAM) with Poisson distribution (see the materials and methods section for GAM constraints). Here, species richness in the climate space was the response variable, with geography of climate and climate itself as predictors. The robustness of our conclusions was tested against different climate space definitions.

We first replaced PET with NPP prior to the PCA in defining the climate space (Supplementary Tables 6 to 9). Next, we modeled a climate space based solely on temperature and precipitation (Supplementary Tables 10 to 13). Lastly, we tested the impact of log-transforming precipitation variables prior to the PCA (Supplementary Tables 14 to 17). The outcomes of these analyses indicate that our conclusions remain robust across these different climate space definitions for terrestrial tetrapods.

However, it's crucial to recognize that defining a space with independent axes can be challenging when using correlated variables (like temperature and precipitation) to represent those axes. This creates a space where the axes are interrelated, given the correlation between average temperature and precipitation. Hence, the concept of a truly two-dimensional space, akin to geographical mapping via longitude and latitude, becomes debatable.

As a solution, we employed a climate space defined by orthogonal principal components from a PCA for our primary results. This approach not only facilitates the inclusion of multiple variables representing energy and water availability constraints but also mathematically enables the establishment of genuinely orthogonal climate axes.

2. Exploring non-linear relationships

In our initial approach, we utilized a Poisson Generalized Linear Model (GLM) with species richness as the response variable, and the geography of climate (area and isolation) and climate itself (PC1 and PC2) as predictors. Although the linear model accounted for approximately 75% of the richness variance for all groups (Supplementary Table 18), the Residual vs Fitted plot indicated non-linearities, violating the assumptions of the linear model and making the estimated parameters uninterpretable (Supplementary Figure 8).

We mitigated these non-linearities with polynomial regression and General Additive Models (GAMs), which both yielded consistent results (Supplementary Tables 1 to 4, Supplementary Tables 19 to 22). The balance with non-linear models, however, is ensuring the right level of model flexibility. A rigid model might neglect the non-linearities, while an overly flexible model could overfit and consequently include data noise. In our polynomial regression, we adjusted the model's flexibility using different polynomial degrees. We evaluated linear GLMs against non-linear polynomial GLMs with varying degrees of flexibility, from two to five. Our conclusions from this approach include: (1) The 4th degree polynomial GLM provided the smallest deviance (Table S19, column "deviance"), effectively addressing the non-linearity issue (Supplementary Fig 8). (2)The 4th degree polynomial GLM demonstrated a goodness-of-fit of about 90% (Supplementary Table 19), comparable to that of a GAM with k = 4. (3) The improvement in goodness-of-fit when moving from a linear to a 4th degree polynomial model was 14%, similar to the improvement seen with the GAM with k = 4. (4) As the 4th degree polynomial GLM doesn't appear to overfit the data and matches the goodness-of-fit of the GAM, we infer that the GAM isn't overfitting either.

We chose to use GAMs in our primary text due to their inherent benefits as extensions of GLMs, providing a safer, simpler alternative to polynomial regressions. GAMs have two main advantages:

Reduced overfitting: GAMs use inherent regularized smoothing functions, aiding in mitigating overfitting, a common issue in polynomial regression.

Robustness to outliers: GAMs, due to the smoothing functions used, are less impacted by extreme data points than polynomial regression models, which can be highly sensitive to outliers.

Overall, this made GAMs a robust choice for our data.

3. Measuring predictors importance

The unique influence of a particular predictor on a model's effectiveness can be determined by measuring the decrease in the model's goodness-of-fit (e.g., proportion of null deviance or adjusted R^2) when that predictor is removed. For instance, our complete model accounts for about 90% of the variance, but this drops to roughly 80% when the 'climate area' predictor is removed. Therefore, 'climate area' uniquely contributes approximately 10% to the model's predictive power. The cumulative unique contributions of all predictors equate to about 30%, indicating that 30% of the explanation is distinctly attributable to a single predictor.

The gap between the full model's explanatory power (90%) and the total unique contributions of all predictors (30%) represents the joint influence (60%) of the predictors.

To provide further insight, we divided this joint contribution into three categories of shared explanation: shared influence within 'geography of climate' (between 'area' and 'isolation' predictors), within 'climate itself' (between 'PC1' and 'PC2' predictors), and across both categories (Fig. 4 of the main text).

The shared explanation within 'geography of climate' and 'climate itself' accounts for approximately 12% and 1-2% respectively. The remaining shared explanation, roughly 45%, is attributable to various combinations of predictors across different categories, for instance, four pairs (e.g., 'area' and 'PC1'), four triplets (e.g., 'area', 'PC1', and 'PC2') and one quadruplet ('area', 'isolation', 'PC1', and 'PC2').

While we could have subdivided the remaining shared explanation among other possible combinations of predictors, we chose to group it under the 'Joint Geography of Climate and Climate Itself' category (Fig 4 of the main text). Further subdivisions wouldn't significantly enhance the interpretation of our results.

The prerequisites for partitioning explanatory power in a model are two-fold: (1) a legitimate statistical measure of the model's goodness-of-fit, and (2) a measure that lies within a defined range, such as the percentage of null deviance or R^2 (in contrast to unbounded measures like SSE or likelihood).

4. Supplementary Figures

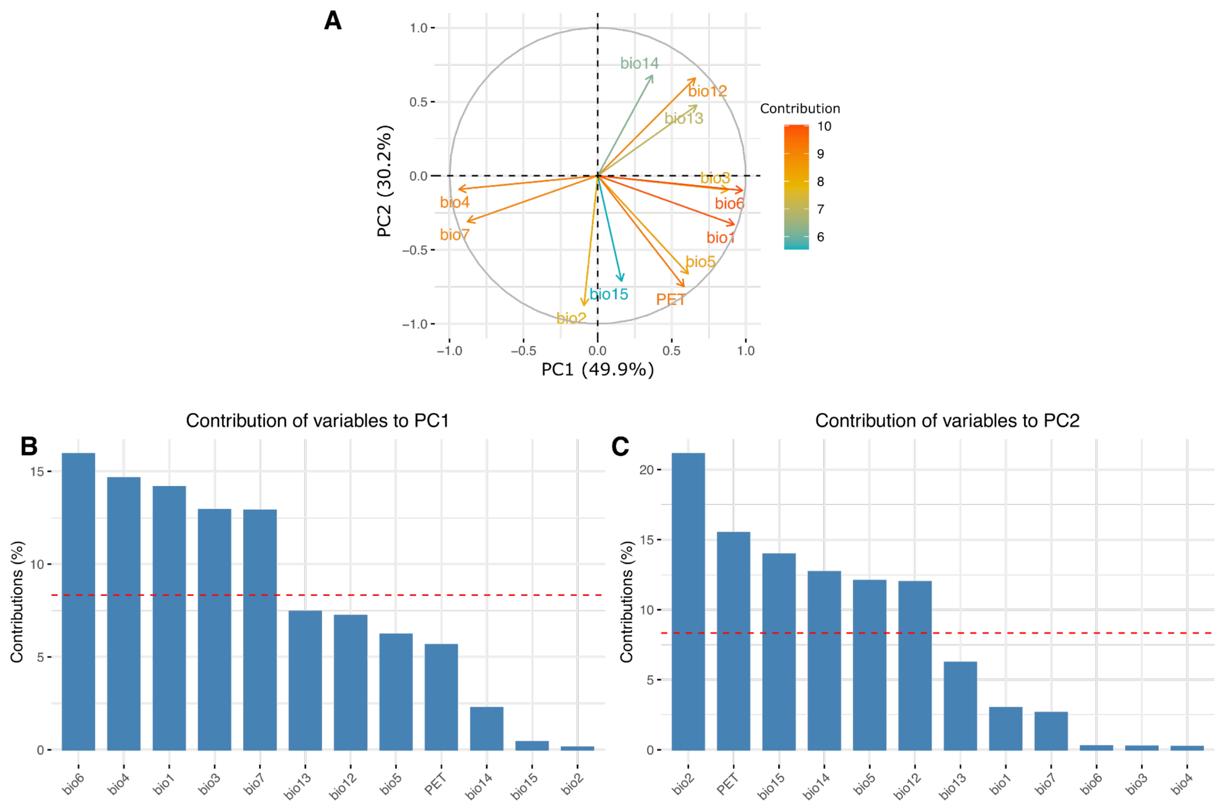

Supplementary Figure 1 | Principal component analysis for twelve climatic variables. **A,** Loading plot showing how strongly each characteristic influences principal components. Each variable is colored based on their squared coordinates, which represents the quality of representation for variables on the factor map. **B, C,** contributions of variables in accounting for the variability in the first two principal components. Red dashed lines indicate the expected contribution of variables if their contributions are uniform. The variables used were: mean annual air temperature (bio1), mean diurnal air temperature range (bio2), isothermality (bio3), temperature seasonality (bio4), mean daily maximum air temperature of the warmest month (bio5), mean daily minimum air temperature of the coldest month (bio6), annual range of air temperature (bio7), annual precipitation amount (bio12), precipitation amount of the wettest month (bio13), precipitation amount of the driest month (bio14), precipitation seasonality (bio15), potential evapotranspiration (PET).

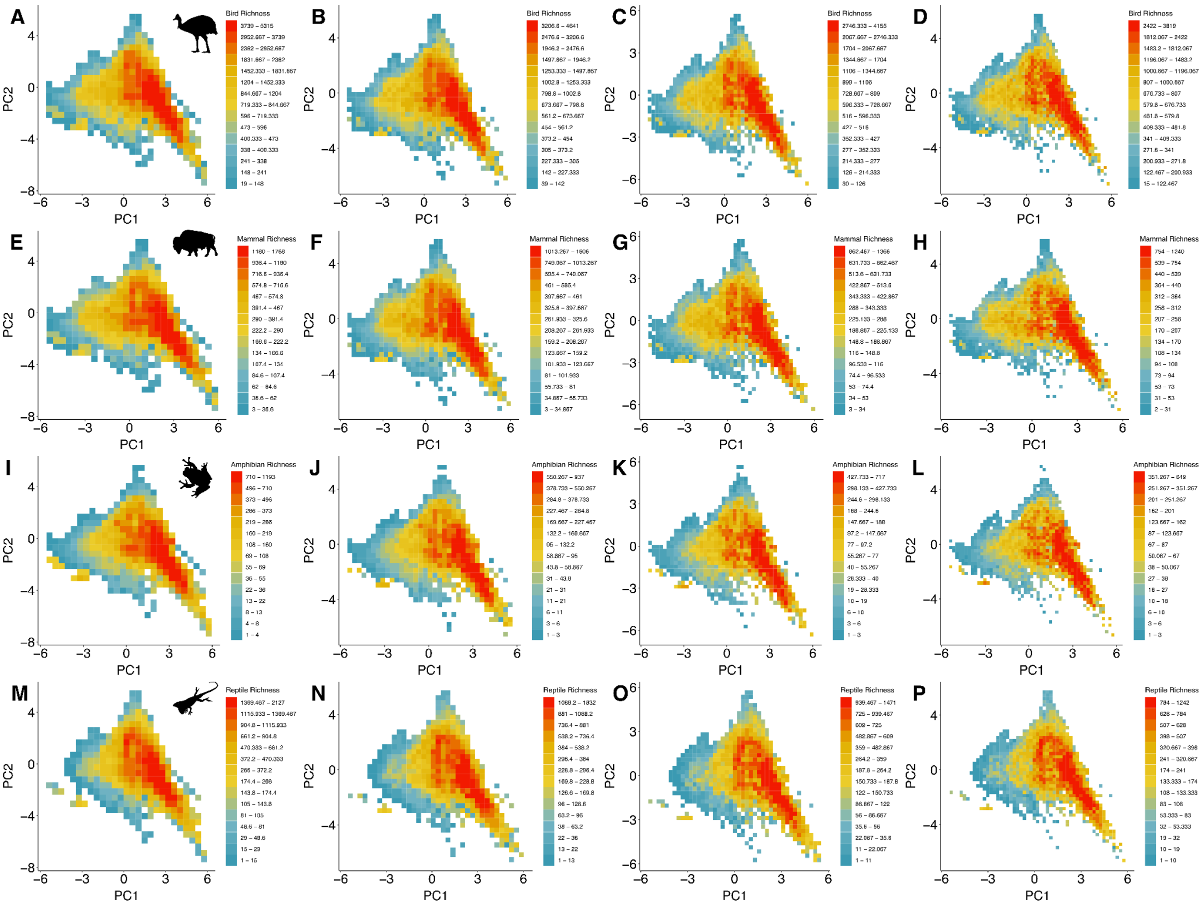

Supplementary Figure 2 | Tetrapod richness in climate space across different resolutions. Each roll represents resolutions of 30, 40, 50 and 60 equal interval divisions of climate space. Number of species that fall within a climatic condition counted for **a** birds, **b** mammals, **c** amphibians and **d** reptiles.

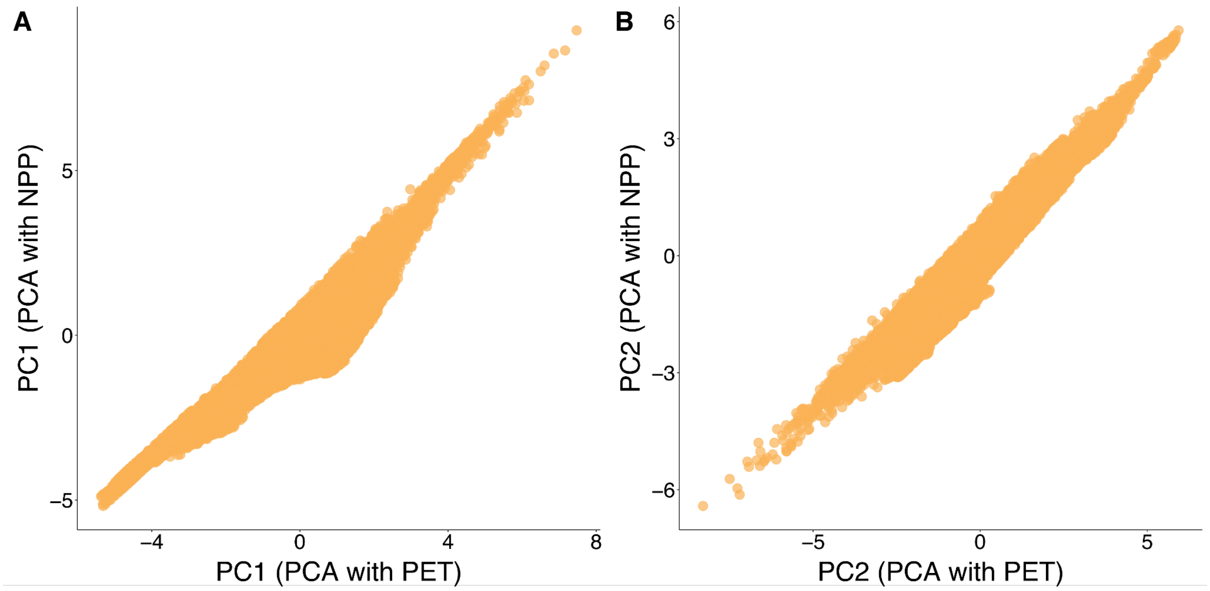

Supplementary Figure 3 | Sensitivity Analysis for the choice of variables in the PCA (n = 13312 geographical cells). Here the first two principal components of two different PCAs are confronted. PC1 and PC2 in y axis represent the scores of a PCA in which PET was replaced by NPP. On the other hand, PC1 and PC2 in the x axis represent the scores of a PCA in which NPP was replaced by PET. **A** PC1 emerging from the two different PCAs are confronted (pearson's r = 0.953) and **B** PC2 emerging from the two different PCAs are confronted (pearson's r = 0.954).

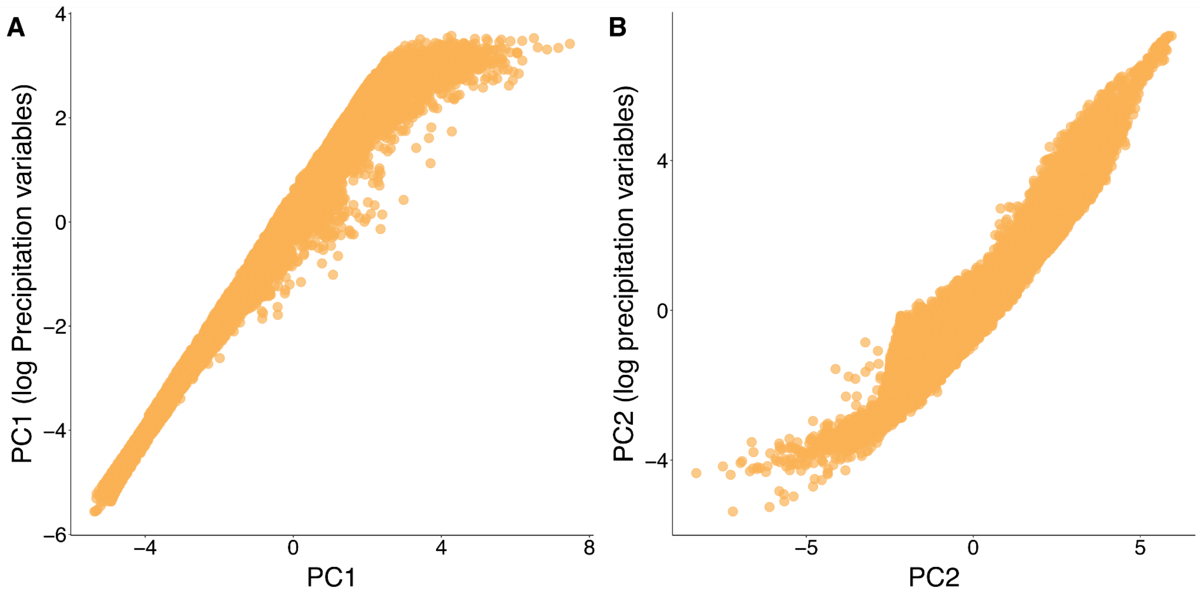

Supplementary Figure 4 | Sensitivity Analysis for the choice of variables in the PCA (n = 13312 geographical cells). Here the first two principal components of two different PCAs are confronted. PC1 and PC2 in y axis represent the scores of a PCA in which precipitation variables were log transformed. On the other hand, PC1 and PC2 in the x axis represent the scores of a PCA in which precipitation variables are in their original scale. **A** PC1 emerging from the two different PCAs are confronted (pearson's r = 0.983) and **B** PC2 emerging from the two different PCAs are confronted (pearson's r = 0.959).

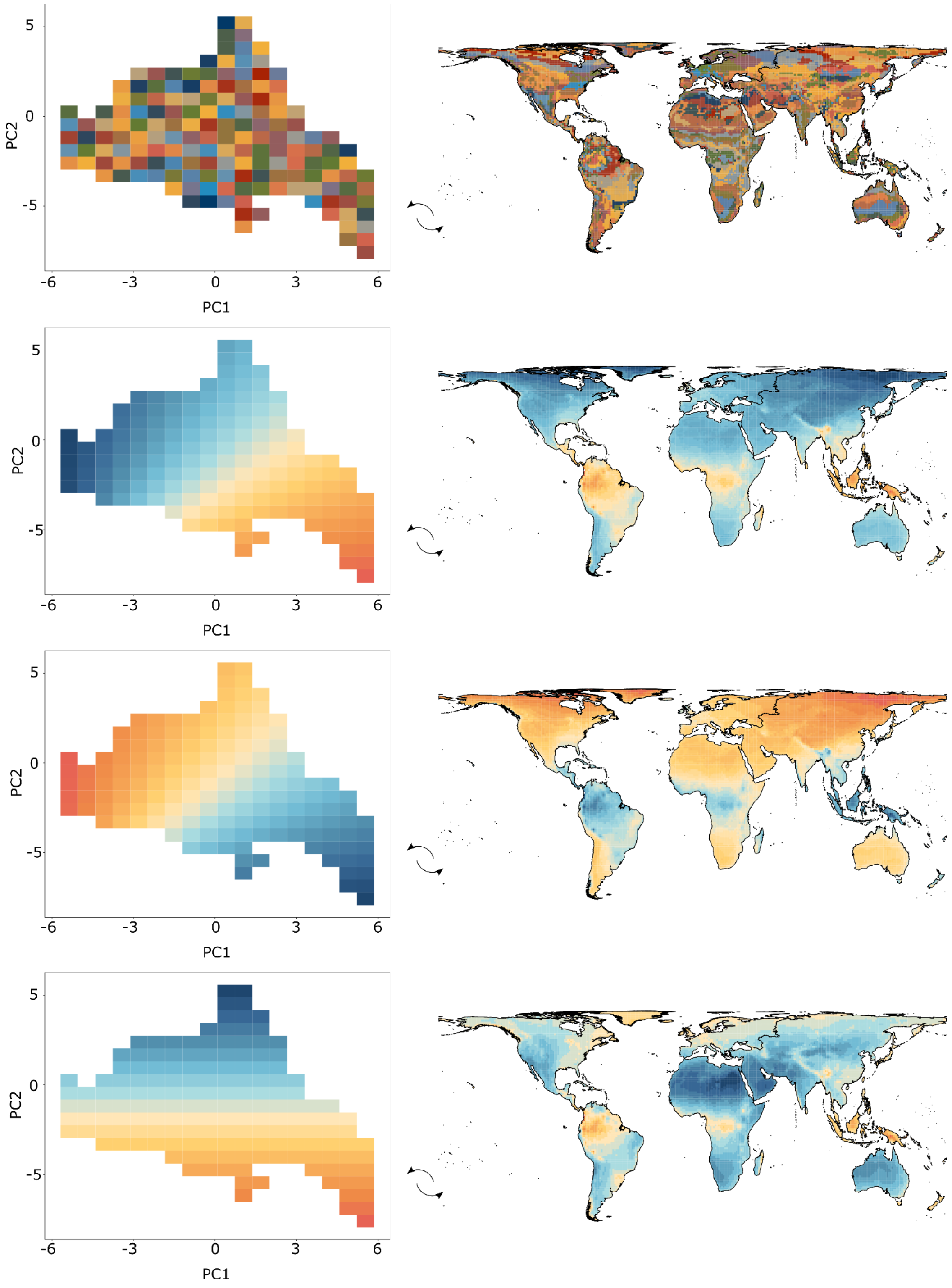

Supplementary Figure 5 | Duality between climate and geographical space. The different figures here represent different color gradients that illustrate the duality between geographical and environmental space. The duality between geographical and climate space refers to the relationship between geographical and climate space: a given climatic condition (climate cell in climate space) is observed in several geographical locations (1 -> n : one climate cell represent n geographical cells) while several geographical locations belong to a unique climate condition (n -> 1 : n geographical cells represent one climate cell). The colors attributed to one climate cell are attributed to several geographical cells and the choice of

which color gradient is used to illustrate the duality concept is arbitrary. Therefore, potential color gradients in climate space do not represent any biological pattern and are an arbitrary choice.

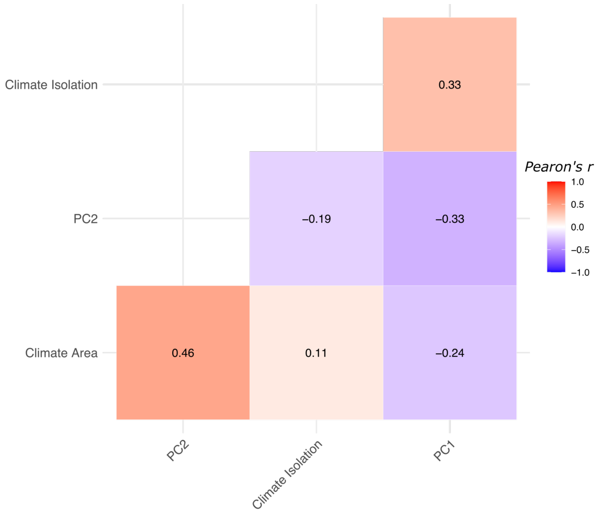

Supplementary Figure 6 | Pairwise correlation among predictor variables. The largest correlation among them is below 0.5, which is much lower than the statistical consensus of potential confounding effects among variables |r| > 0.7

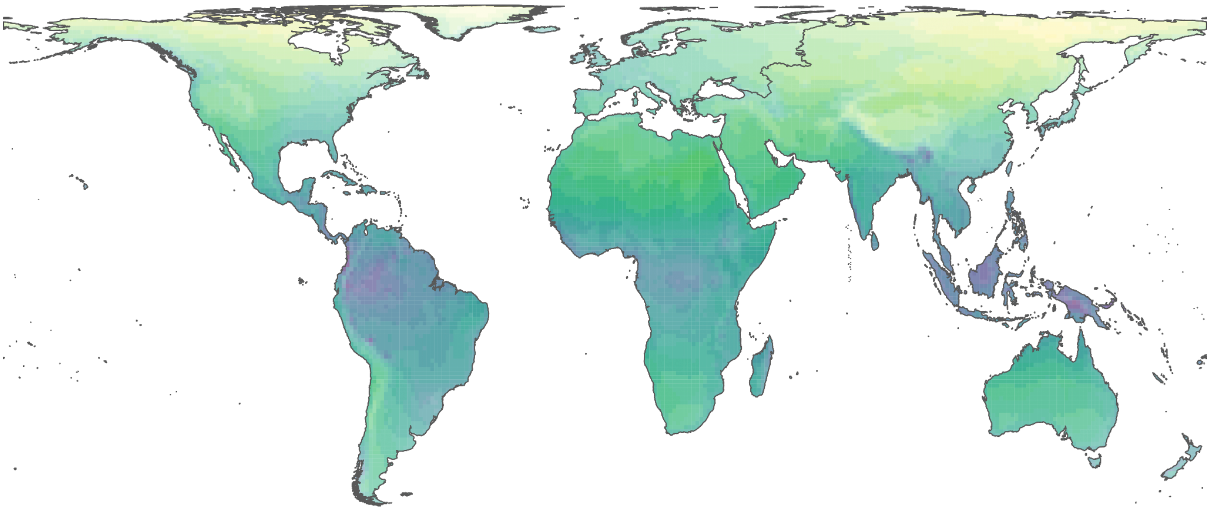

Supplementary Figure 7 | Duality colors of Fig 1d in a larger map.

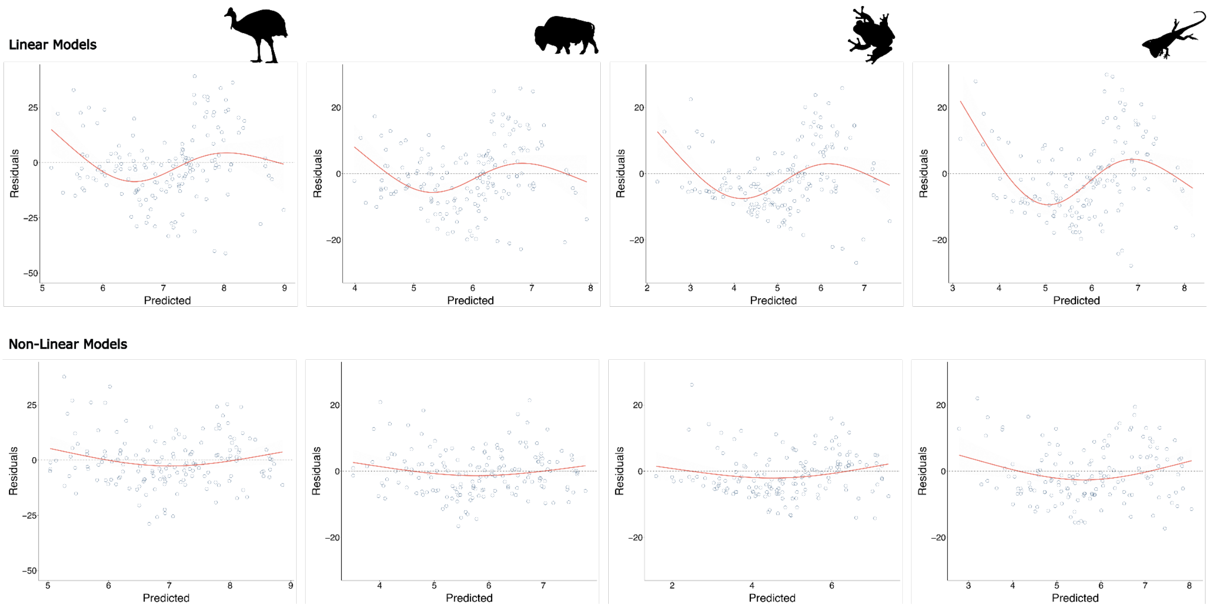

Supplementary Figure 8 | Evaluation of Linear and Polynomial GLM Fit Using Residual vs Fitted Plot. This visualization represents the residuals (the gaps between observed and predicted values) on the y-axis plotted against the fitted (predicted) values on the x-axis. The analysis includes both the linear Generalized Linear Model (GLM) and the four-degree polynomial GLM. The plot's purpose is to assess the quality of the model's fit and to spot potential non-linearity and heteroscedasticity within the data. Ideally, for a well-fitted model that meets linearity and homoscedasticity assumptions, residuals should scatter randomly around the horizontal zero line across the fitted values range. Any systematic deviations from this line suggest issues with the model fit and breaches of model assumptions. Linear models in this case violate the linearity assumption, but once non-linear models are assumed, the residuals no longer present systematic trends.

Supplementary Tables

Supplementary Table 1 | Comparative Analysis of Predictor Impact on Bird Richness Patterns Using General Additive Models Across Multiple Climate Space Intervals. The same predictors as in Extended Data Table 1 —climate area, climate isolation (components of geography of climate), and principal components one and two (components of climate itself)—are utilized in these models. The difference here is that analyses were performed on gridded climate spaces defined with 30, 40, 50, and 60 equal intervals along the climate axis. As with Supplementary Table S1, each predictor's importance is assessed by the reduction in deviance when it is excluded from the full model. The collective influence of the predictors is similarly divided into three dimensions: (i) the joint effect of geography of climate elements, (ii) the joint effect of climate itself elements, and (iii) the integrated effect of both geography of climate and climate itself elements. The p-values for each variable are computed using two-sided tests.

Birds (30 equal interval resolution) *Adjusted R^2 = 0.91, Predicted R^2 = 0.92*

	Proportion of Null Deviance	VIF	Chi.sq	p-value
Climate Area	0.07	1.33	26603	<2E-16
Climate Isolation	0.08	1.19	21624	<2E-16
PC1	0.13	1.22	53619	<2E-16
PC2	0.02	1.34	9216	<2E-16
Joint Contribution within the geography of climate	0.12			
Joint Contribution within climate itself	0.02			
Joint Contribution between the geography of climate and climate itself	0.47			
Total	0.91			

Birds (40 equal interval resolution) *Adjusted R^2 = 0.91, Predicted R^2 = 0.91*

	Proportion of Null Deviance	VIF	Chi.sq	p-value
Climate Area	0.07	1.27	36835	<2E-16
Climate Isolation	0.07	1.20	22586	<2E-16
PC1	0.13	1.19	69394	<2E-16
PC2	0.02	1.25	13239	<2E-16
Joint Contribution within the geography of climate	0.11			
Joint Contribution within climate itself	0.02			
Joint Contribution between the geography of climate and climate itself	0.48			
Total	0.90			

Birds (50 equal interval resolution) *Adjusted R^2 = 0.89, Predicted R^2 = 0.90*

	Proportion of Null Deviance	VIF	Chi.sq	p-value
Climate Area	0.08	1.21	47268	<2E-16
Climate Isolation	0.06	1.22	20822	<2E-16
PC1	0.15	1.19	100394	<2E-16
PC2	0.03	1.19	19269	<2E-16
Joint Contribution within the geography of climate	0.13			
Joint Contribution within climate itself	0.02			
Joint Contribution between the geography of climate and climate itself	0.42			
Total	0.89			

Birds (60 equal interval resolution) - *Adjusted R^2 = 0.89, Predicted R^2 = 0.89*

	Proportion of Null Deviance	VIF	Chi.sq	p-value
Climate Area	0.08	1.19	54357	<2E-16
Climate Isolation	0.05	1.21	22019	<2E-16
PC1	0.16	1.19	119614	<2E-16
PC2	0.03	1.16	26036	<2E-16
Joint Contribution within the geography of climate	0.13			
Joint Contribution within climate itself	0.03			
Joint Contribution between the geography of climate and climate itself	0.4			
Total	0.88			

Supplementary Table 2 | Comparative Analysis of Predictor Impact on Mammal Richness Patterns Using General Additive Models Across Multiple Climate Space Intervals. The same predictors as in Supplementary Table 1—climate area, climate isolation (components of geography of climate), and principal components one and two (components of climate itself)—are utilized in these models. The difference here is that analyses were performed on gridded climate spaces defined with 30, 40, 50, and 60 equal intervals along the climate axis. As with Supplementary Table 1, each predictor's importance is assessed by the reduction in deviance when it is excluded from the full model. The collective influence of the predictors is similarly divided into three dimensions: (i) the joint effect of geography of climate elements, (ii) the joint effect of climate itself elements, and (iii) the integrated effect of both geography of climate and climate itself elements. The p-values for each variable are computed using two-sided tests.

Mammals (30 equal interval resolution) - *Adjusted R^2 = 0.89, Predicted R^2 = 0.90*

	**Proportion of Null Deviance**	**VIF**	**Chi.sq**	**p-value**
Climate Area	0.09	1.33	10815	<2E-16
Climate Isolation	0.07	1.19	5934	<2E-16
PC1	0.12	1.22	17548	<2E-16
PC2	0.03	1.32	3965	<2E-16
Joint Contribution within the geography of climate	0.12			
Joint Contribution within climate itself	0.02			
Joint Contribution between the geography of climate and climate itself	0.44			
Total	0.89			

Mammals (40 equal interval resolution) - *Adjusted R^2 = 0.88, Predicted R^2 = 0.89*

	**Proportion of Null Deviance**	**VIF**	**Chi.sq**	**p-value**
Climate Area	0.10	1.27	14793	<2E-16
Climate Isolation	0.05	1.20	5659	<2E-16
PC1	0.12	1.19	23424	<2E-16
PC2	0.03	1.25	5577	<2E-16
Joint Contribution within the geography of climate	0.12			
Joint Contribution within climate itself	0.02			
Joint Contribution between the geography of climate and climate itself	0.44			
Total	0.88			

Mammals (50 equal interval resolution) - *Adjusted R^2 = 0.88, Predicted R^2 = 0.88*

	Proportion of Null Deviance	VIF	Chi.sq	p-value
Climate Area	0.09	1.21	14793	<2E-16
Climate Isolation	0.05	1.22	5659	<2E-16
PC1	0.15	1.19	23424	<2E-16
PC2	0.03	1.19	5577	<2E-16
Joint Contribution within the geography of climate	0.14			
Joint Contribution within climate itself	0.02			
Joint Contribution between the geography of climate and climate itself	0.39			
Total	0.87			

Mammals (60 equal interval resolution) - *Adjusted R^2 = 0.87, Predicted R^2 = 0.87*

	Proportion of Null Deviance	VIF	Chi.sq	p-value
Climate Area	0.11	1.19	21009	<2E-16
Climate Isolation	0.02	1.21	5085	<2E-16
PC1	0.19	1.19	39858	<2E-16
PC2	0.06	1.16	10349	<2E-16
Joint Contribution within the geography of climate	0.08			
Joint Contribution within climate itself	0.03			
Joint Contribution between the geography of climate and climate itself	0.37			
Total	0.87			

Supplementary Table 3 | Comparative Analysis of Predictor Impact on Amphibian Richness Patterns Using General Additive Models Across Multiple Climate Space Intervals. The same predictors as in Supplementary Table 1—climate area, climate isolation (components of geography of climate), and principal components one and two (components of climate itself)—are utilized in these models. The difference here is that analyses were performed on gridded climate spaces defined with 30, 40, 50, and 60 equal intervals along the climate axis. As with Supplementary Table 1, each predictor's importance is assessed by the reduction in deviance when it is excluded from the full model. The collective influence of the predictors is similarly divided into three dimensions: (i) the joint effect of geography of climate elements, (ii) the joint effect of climate itself elements, and (iii) the integrated effect of both geography of climate and climate itself elements. The p-values for each variable are computed using two-sided tests.

Amphibians (30 equal interval resolution) - *Adjusted R^2 = 0.87, Predicted R^2 = 0.87*

	Proportion of Null Deviance	VIF	Chi.sq	p-value
Climate Area	0.08	1.33	6856	<2E-16
Climate Isolation	0.07	1.16	3711	<2E-16
PC1	0.16	1.21	15225	<2E-16
PC2	0.02	1.34	2153	<2E-16
Joint Contribution within the geography of climate	0.11			
Joint Contribution within climate itself	0.01			
Joint Contribution between the geography of climate and climate itself	0.41			
Total	0.86			

Amphibians (40 equal interval resolution) - *Adjusted R^2 = 0.86, Predicted R^2 = 0.86*

	Proportion of Null Deviance	VIF	Chi.sq	p-value
Climate Area	0.07	1.27	7790	<2E-16
Climate Isolation	0.06	1.16	4466	<2E-16
PC1	0.16	1.18	17136	<2E-16
PC2	0.02	1.28	2282	<2E-16
Joint Contribution within the geography of climate	0.11			
Joint Contribution within climate itself	0.00			
Joint Contribution between the geography of climate and climate itself	0.43			
Total	0.85			

Amphibians (50 equal interval resolution) - *Adjusted R^2 = 0.84, Predicted R^2 = 0.84*

	Proportion of Null Deviance	VIF	Chi.sq	p-value
Climate Area	0.07	1.22	9047	<2E-16
Climate Isolation	0.05	1.16	3750	<2E-16
PC1	0.19	1.17	23006	<2E-16
PC2	0.03	1.21	3708	<2E-16
Joint Contribution within the geography of climate	0.13			
Joint Contribution within climate itself	0			
Joint Contribution between the geography of climate and climate itself	0.36			
Total	0.83			

Amphibians (60 equal interval resolution) - *Adjusted R^2 = 0.82, Predicted R^2 = 0.82*

	Proportion of Null Deviance	VIF	Chi.sq	p-value
Climate Area	0.06	1.19	8421	<2E-16
Climate Isolation	0.04	1.16	3994	<2E-16
PC1	0.20	1.17	26225	<2E-16
PC2	0.03	1.18	4921	<2E-16
Joint Contribution within the geography of climate	0.13			
Joint Contribution within climate itself	0.01			
Joint Contribution between the geography of climate and climate itself	0.35			
Total	0.82			

Supplementary Table 4 | Comparative Analysis of Predictor Impact on Reptile Richness Patterns Using General Additive Models Across Multiple Climate Space Intervals. The same predictors as in Supplementary Table 1—climate area, climate isolation (components of geography of climate), and principal components one and two (components of climate itself)—are utilized in these models. The difference here is that analyses were performed on gridded climate spaces defined with 30, 40, 50, and 60 equal intervals along the climate axis. As with Supplementary Table 1, each predictor's importance is assessed by the reduction in deviance when it is excluded from the full model. The collective influence of the predictors is similarly divided into three dimensions: (i) the joint effect of geography of climate elements, (ii) the joint effect of climate itself elements, and (iii) the integrated effect of both geography of climate and climate itself elements. The p-values for each variable are computed using two-sided tests.

Reptiles (30 equal interval resolution) - *Adjusted R^2 = 0.90, Predicted R^2 = 0.89*

	**Proportion of Null Deviance**	**VIF**	**Chi.sq**	**p-value**
Climate Area	0.08	1.32	13225	<2E-16
Climate Isolation	0.07	1.12	7990	<2E-16
PC1	0.16	1.21	26968	<2E-16
PC2	0.02	1.37	3126	7.36E-05
Joint Contribution within the geography of climate	0.11			
Joint Contribution within climate itself	0.01			
Joint Contribution between the geography of climate and climate itself	0.41			
Total	0.86			

Reptiles (40 equal interval resolution) - *Adjusted R^2 = 0.89, Predicted R^2 = 0.89*

	**Proportion of Null Deviance**	**VIF**	**Chi.sq**	**p-value**
Climate Area	0.07	1.25	16260	<2E-16
Climate Isolation	0.06	1.14	9303	<2E-16
PC1	0.16	1.17	34072	<2E-16
PC2	0.02	1.30	3632	<2E-16
Joint Contribution within the geography of climate	0.11			
Joint Contribution within climate itself	0			
Joint Contribution between the geography of climate and climate itself	0.43			
Total	0.85			

Reptiles (50 equal interval resolution) - *Adjusted R^2 = 0.88, Predicted R^2 = 0.88*

	Proportion of Null Deviance	VIF	Chi.sq	p-value
Climate Area	0.07	1.19	19285	<2E-16
Climate Isolation	0.05	1.14	8400	<2E-16
PC1	0.19	1.16	46271	<2E-16
PC2	0.03	1.23	4261	<2E-16
Joint Contribution within the geography of climate	0.13			
Joint Contribution within climate itself	0.00			
Joint Contribution between the geography of climate and climate itself	0.36			
Total	0.83			

Reptiles (60 equal interval resolution) - *Adjusted R^2 = 0.88, Predicted R^2 = 0.87*

	Proportion of Null Deviance	VIF	Chi.sq	p-value
Climate Area	0.06	1.16	20948	<2E-16
Climate Isolation	0.04	1.13	8797	<2E-16
PC1	0.20	1.15	55186	<2E-16
PC2	0.03	1.20	5632	<2E-16
Joint Contribution within the geography of climate	0.13			
Joint Contribution within climate itself	0.01			
Joint Contribution between the geography of climate and climate itself	0.35			
Total	0.82			

Supplementary Table 5 | PCA loadings. The variables used in the PCA were: mean annual air temperature (bio1), mean diurnal air temperature range (bio2), isothermality (bio3), temperature seasonality (bio4), mean daily maximum air temperature of the warmest month (bio5), mean daily minimum air temperature of the coldest month (bio6), annual range of air temperature (bio7), annual precipitation amount (bio12), precipitation amount of the wettest month (bio13), precipitation amount of the driest month (bio14), precipitation seasonality (bio15), potential evapotranspiration (PET).

	PC1	PC2	PC3	PC4	PC5	PC6	PC7	PC8	PC9	PC10	PC11	PC12
**Bio1**	0.3763	-0.1733	0.1154	0.0270	0.2466	0.0634	-0.1016	-0.2067	-0.0257	0.6947	-0.4618	0.0000
**Bio2**	-0.0377	-0.4596	0.2665	-0.2068	-0.3639	-0.4189	0.4616	-0.3230	0.0477	0.1256	0.1738	-9E-07
**Bio3**	0.3595	-0.0504	-0.0039	0.0692	-0.5409	-0.4025	-0.5557	0.3039	-0.0847	0.0146	-0.0162	-8E-06
**Bio4**	-0.3827	-0.0480	0.1703	-0.3085	0.1335	0.0587	-0.3230	0.2468	0.0028	0.5067	0.5327	7E-06
**Bio5**	0.2496	-0.3475	0.3203	-0.2039	0.4003	0.0046	-0.3496	-0.2034	-0.0298	-0.4386	0.1262	4E-01
**Bio6**	0.3993	-0.0523	0.0084	0.1729	0.1633	0.0656	-0.0273	-0.1663	-0.0009	-0.0578	0.4469	-7E-01
**Bio7**	-0.3592	-0.1627	0.2024	-0.3634	0.0518	-0.0833	-0.1970	0.0833	-0.0188	-0.2161	-0.5042	-6E-01
**Bio12**	0.2689	0.3463	0.0465	-0.4116	0.0034	-0.0746	0.0441	0.0417	0.7917	-0.0057	-0.0127	-1E-05
**Bio13**	0.2730	0.2498	-0.2730	-0.5535	0.2235	-0.2880	0.2351	0.1430	-0.5219	0.0076	0.0328	5E-06
**Bio14**	0.1506	0.3564	0.5518	-0.1969	-0.4003	0.4955	0.0438	-0.1230	-0.2895	-0.0205	0.0003	2E-07
**Bio15**	0.0658	-0.3737	-0.5781	-0.3711	-0.3071	0.4918	-0.1250	-0.1723	0.0461	-0.0094	0.0190	-1E-06
**PET**	0.2379	-0.3935	0.1664	0.0263	0.0467	0.2605	0.3563	0.7461	0.0596	-0.0418	-0.0332	-1E-06

Supplementary Table 6 | Observed statistics of general additive models for birds when models are fitted assuming different resolutions of climate space. Models were fitted assuming the richness patterns of birds as response variable and climate area, climate isolation and principal components one and two as predictors. Here NPP replaces PET in the PCA as explained in *Sensitivity analysis for different definitions of the climate space* section**.** The gridded climate space is defined based on 20, 30, 40, 50 and 60 equal interval divisions of the climate axis. As with Supplementary Table 1, each predictor's importance is assessed by the reduction in deviance when it is excluded from the full model. The p-values for each variable are computed using two-sided tests.

***Birds (20 equal interval resolution)** - Adjusted R^2 = 0.90, Predicted R^2 =0.90*				
	**Proportion of Null Deviance**	**VIF**	**Chi.sq**	**p-value**
Climate Area	0.08	1.36	19851	<2E-16
Climate Isolation	0.08	1.24	11937	<2E-16
PC1	0.13	1.29	34057	<2E-16
PC2	0.02	1.31	4631	<2E-16
Joint Contribution within the geography of climate	0.09			
Joint Contribution within climate itself	0.02			
Joint Contribution between the geography of climate and climate itself	0.48			
Total	0.90			
***Birds (30 equal interval resolution)** - Adjusted R^2 = 0.89, Predicted R^2 = 0.89*				
Climate Area	0.06	1.30	25679	<2E-16
Climate Isolation	0.07	1.14	19102	<2E-16
PC1	0.15	1.21	61303	<2E-16
PC2	0.02	1.28	8680	<2E-16
Joint Contribution within the geography of climate	0.11			
Joint Contribution within climate itself	0.02			
Joint Contribution between the geography of climate and climate itself	0.46			
Total	0.89			
**Birds (40 equal interval resolution)** - *Adjusted R^2 = 0.89, Predicted R^2 = 0.89*				
Climate Area	0.06	1.27	47268	<2E-16
Climate Isolation	0.07	1.15	20822	<2E-16
PC1	0.17	1.19	100394	<2E-16
PC2	0.02	1.27	19269	<2E-16

Joint Contribution within the geography of climate	0.13			
Joint Contribution within climate itself	0.01			
Joint Contribution between the geography of climate and climate itself	0.43			
Total	0.90			
**Birds (50 equal interval resolution)** - *Adjusted R^2 = 0.88, Predicted R^2 = 0.88*				
Climate Area	0.07	1.23	41858	<2E-16
Climate Isolation	0.07	1.18	28197	<2E-16
PC1	0.17	1.19	108446	<2E-16
PC2	0.03	1.21	18702	<2E-16
Joint Contribution within the geography of climate	0.13			
Joint Contribution within climate itself	0.01			
Joint Contribution between the geography of climate and climate itself	0.40			
Total	0.88			
**Birds (60 equal interval resolution)** - *Adjusted R^2 = 0.88, Predicted R^2 = 0.87*				
Climate Area	0.08	1.20	53361	<2E-16
Climate Isolation	0.05	1.16	22975	<2E-16
PC1	0.18	1.16	135352	<2E-16
PC2	0.03	1.17	25719	<2E-16
Joint Contribution within the geography of climate	0.14			
Joint Contribution within climate itself	0.03			
Joint Contribution between the geography of climate and climate itself	0.36			
Total	0.87			

Supplementary Table 7 | Observed statistics of general additive models for mammals when models are fitted assuming different resolutions of climate space. Models were fitted assuming the richness patterns of mammals as response variable and climate area, climate isolation and principal components one and two as predictors. Here NPP replaces PET in the PCA as explained in *Sensitivity analysis for different definitions of the climate space* section**.** The gridded climate space is defined based on 20, 30, 40, 50 and 60 equal interval divisions of the climate axis. As with Supplementary Table 1, each predictor's importance is assessed by the reduction in deviance when it is excluded from the full model. The p-values for each variable are computed using two-sided tests.

Mammals (20 equal interval resolution) *- Adjusted R^2 = 0.89, Predicted R^2 = 0.89*				
	**Proportion of Null Deviance**	**VIF**	**Chi.sq**	**p-value**
Climate Area	0.09	1.36	8259	<2E-16
Climate Isolation	0.07	1.24	3355	<2E-16
PC1	0.11	1.29	10520	<2E-16
PC2	0.02	1.31	2090	<2E-16
Joint Contribution within the geography of climate	0.10			
Joint Contribution within climate itself	0.02			
Joint Contribution between the geography of climate and climate itself	0.48			
Total	0.89			
Mammals (30 equal interval resolution) *- Adjusted R^2 = 0.87, Predicted R^2 = 0.87*				
Climate Area	0.08	1.30	9910	<2E-16
Climate Isolation	0.07	1.14	5740	<2E-16
PC1	0.13	1.21	18657	<2E-16
PC2	0.02	1.28	3531	<2E-16
Joint Contribution within the geography of climate	0.11			
Joint Contribution within climate itself	0.02			
Joint Contribution between the geography of climate and climate itself	0.45			
Total	0.88			
**Mammals (40 equal interval resolution)** *- Adjusted R^2 = 0.87, Predicted R^2 = 0.87*				
Climate Area	0.08	1.27	12878	<2E-16
Climate Isolation	0.05	1.16	6094	<2E-16
PC1	0.16	1.19	28204	<2E-16

PC2	0.02	1.26	4791	<2E-16
Joint Contribution within the geography of climate	0.14			
Joint Contribution within climate itself	0.02			
Joint Contribution between the geography of climate and climate itself	0.40			
Total	0.87			
**Mammals (50 equal interval resolution)** - *Adjusted R^2 = 0.86, Predicted R^2 = 0.86*				
Climate Area	0.09	1.23	16466	<2E-16
Climate Isolation	0.06	1.19	7463	<2E-16
PC1	0.16	1.20	35678	<2E-16
PC2	0.03	1.21	7136	<2E-16
Joint Contribution within the geography of climate	0.13			
Joint Contribution within climate itself	0.02			
Joint Contribution between the geography of climate and climate itself	0.37			
Total	0.85			
**Mammals (60 equal interval resolution)** - *Adjusted R^2 = 0.85, Predicted R^2 = 0.84*				
Climate Area	0.08	1.20	20320	<2E-16
Climate Isolation	0.04	1.16	6059	<2E-16
PC1	0.18	1.16	44548	<2E-16
PC2	0.04	1.16	9381	<2E-16
Joint Contribution within the geography of climate	0.15			
Joint Contribution within climate itself	0.03			
Joint Contribution between the geography of climate and climate itself	0.33			
Total	0.85			

Supplementary Table 8 | Observed statistics of general additive models for amphibians when models are fitted assuming different resolutions of climate space. Models were fitted assuming the richness patterns of reptiles as response variable and climate area, climate isolation and principal components one and two as predictors. Here NPP replaces PET in the PCA as explained in *Sensitivity analysis for different definitions of the climate space* section**.** The gridded climate space is defined based on 20, 30, 40, 50 and 60 equal interval divisions of the climate axis. As with Supplementary Table 1, each predictor's importance is assessed by the reduction in deviance when it is excluded from the full model. The p-values for each variable are computed using two-sided tests.

Amphibians (20 equal interval resolution) *- Adjusted R^2 = 0.87, Predicted R^2 = 0.87*				
	**Proportion of Null Deviance**	**VIF**	**Chi.sq**	**p-value**
Climate Area	0.09	1.36	6302.2	<2E-16
Climate Isolation	0.07	1.20	2427.5	<2E-16
PC1	0.15	1.30	10081.6	<2E-16
PC2	0.01	1.36	904.9	<2E-16
Joint Contribution within the geography of climate	0.09			
Joint Contribution within climate itself	0.01			
Joint Contribution between the geography of climate and climate itself	0.43			
Total	0.85			
Amphibians(30 equal interval resolution) *- Adjusted R^2 = 0.83, Predicted R^2 = 0.83*				
Climate Area	0.07	1.30	6459	<2E-16
Climate Isolation	0.07	1.10	3644	<2E-16
PC1	0.18	1.22	15924	<2E-16
PC2	0.02	1.32	1430	<2E-16
Joint Contribution within the geography of climate	0.10			
Joint Contribution within climate itself	0.00			
Joint Contribution between the geography of climate and climate itself	0.40			
Total	0.84			
***Amphibians*(40 equal interval resolution)** *- Adjusted R^2 = 0.82, Predicted R^2 = 0.82*				
Climate Area	0.07	1.28	7339	<2E-16
Climate Isolation	0.05	1.13	3361	<2E-16
PC1	0.2	1.19	20585	<2E-16

PC2	0.02	1.30	1867	<2E-16
Joint Contribution within the geography of climate	0.12			
Joint Contribution within climate itself	0.00			
Joint Contribution between the geography of climate and climate itself	0.36			
Total	0.81			
***Amphibians*(50 equal interval resolution)** - *Adjusted R^2 = 0.80, Predicted R^2 = 0.80*				
Climate Area	0.06	1.24	7508	<2E-16
Climate Isolation	0.06	1.15	3999	<2E-16
PC1	0.21	1.21	24105	<2E-16
PC2	0.02	1.25	2965	<2E-16
Joint Contribution within the geography of climate	0.12			
Joint Contribution within climate itself	0.01			
Joint Contribution between the geography of climate and climate itself	0.33			
Total	0.81			
***Amphibians* (60 equal interval resolution)** - *Adjusted R^2 = 0.79, Predicted R^2 = 0.76*				
Climate Area	0.07	1.19	9299	<2E-16
Climate Isolation	0.04	1.14	3277	<2E-16
PC1	0.23	1.16	28278	<2E-16
PC2	0.03	1.19	4508	<2E-16
Joint Contribution within the geography of climate	0.13			
Joint Contribution within climate itself	0.0			
Joint Contribution between the geography of climate and climate itself	0.30			
Total	0.8			

Supplementary Table 9 | Observed statistics of general additive models for reptiles when models are fitted assuming different resolutions of climate space. Models were fitted assuming the richness patterns of reptiles as response variable and climate area, climate isolation and principal components one and two as predictors. Here NPP replaces PET in the PCA as explained in *Sensitivity analysis for different definitions of the climate space* section**.** The gridded climate space is defined based on 20, 30, 40, 50 and 60 equal interval divisions of the climate axis. As with Supplementary Table 1, each predictor's importance is assessed by the reduction in deviance when it is excluded from the full model. The p-values for each variable are computed using two-sided tests.

Reptiles (20 equal interval resolution) *- Adjusted R^2 = 0.90, Predicted R^2 = 0.90*				
	**Proportion of Null Deviance**	**VIF**	**Chi.sq**	**p-value**
Climate Area	0.09	1.34	10754	<2E-16
Climate Isolation	0.06	1.21	4706	<2E-16
PC1	0.16	1.28	18219	<2E-16
PC2	0.02	1.39	2090	<2E-16
Joint Contribution within the geography of climate	0.07			
Joint Contribution within climate itself	0.00			
Joint Contribution between the geography of climate and climate itself	0.49			
Total	0.89			
Reptiles (30 equal interval resolution) *- Adjusted R^2 = 0.89, Predicted R^2 = 0.88*				
Climate Area	0.06	1.27	11329	<2E-16
Climate Isolation	0.06	1.10	8085	<2E-16
PC1	0.19	1.23	28507	<2E-16
PC2	0.02	1.36	3116	<2E-16
Joint Contribution within the geography of climate	0.09			
Joint Contribution within climate itself	0.01			
Joint Contribution between the geography of climate and climate itself	0.46			
Total	0.89			
**Reptiles (40 equal interval resolution)** *- Adjusted R^2 = 0.88, Predicted R^2 = 0.87*				
Climate Area	0.06	1.25	13371	<2E-16
Climate Isolation	0.06	1.09	9361	<2E-16
PC1	0.22	1.19	39551	<2E-16
PC2	0.02	1.34	3824	<2E-16

Joint Contribution within the geography of climate	0.10			
Joint Contribution within climate itself	0.00			
Joint Contribution between the geography of climate and climate itself	0.41			
Total	0.87			
**Reptiles (50 equal interval resolution)** - *Adjusted R^2 = 0.87, Predicted R^2 = 0.86*				
Climate Area	0.06	1.24	17788	<2E-16
Climate Isolation	0.06	1.15	10391	<2E-16
PC1	0.24	1.21	48185	<2E-16
PC2	0.02	1.25	4573	<2E-16
Joint Contribution within the geography of climate	0.10			
Joint Contribution within climate itself	0.01			
Joint Contribution between the geography of climate and climate itself	0.38			
Total	0.87			
**Reptiles (60 equal interval resolution)** - *Adjusted R^2 = 0.86, Predicted R^2 = 0.86*				
Climate Area	0.07	1.17	21354	<2E-16
Climate Isolation	0.04	1.11	9417	<2E-16
PC1	0.26	1.14	56735	<2E-16
PC2	0.02	1.22	5871	<2E-16
Joint Contribution within the geography of climate	0.11			
Joint Contribution within climate itself	0.01			
Joint Contribution between the geography of climate and climate itself	0.35			
Total	0.85			

Supplementary Table 10 | Observed statistics of general additive models for birds when models are fitted assuming different resolutions of climate space. Models were fitted assuming the richness patterns of birds as response variable and climate area, climate isolation, **mean annual temperature** and **mean annual precipitation** as predictors. Here climate space is defined by ***temperature (Bio1)*** and ***precipitation (Bio12)*** as explained in *Sensitivity analysis for different definitions of the climate space* section. The gridded climate space is defined based on 20, 30, 40, 50 and 60 equal interval divisions of the climate axis. As with Supplementary Table 1, each predictor's importance is assessed by the reduction in deviance when it is excluded from the full model. The p-values for each variable are computed using two-sided tests.

	Proportion of Null Deviance	VIF	Chi.sq	p-value
***Birds (20 equal interval resolution)** - Adjusted R^2 = 0.92, Predicted R^2 = 0.93*				
Climate Area	0.04	2.14	9124	<2E-16
Climate Isolation	0.07	1.35	7667	<2E-16
Bio1	0.12	1.46	31947	<2E-16
Bio12	0.03	2.62	8166	<2E-16
Joint Contribution within the geography of climate	0.06			
Joint Contribution within climate itself	0.03			
Joint Contribution between the geography of climate and climate itself	0.57			
Total	0.92			
***Birds (30 equal interval resolution)** - Adjusted R^2 = 0.92, Predicted R^2 = 0.92*				
Climate Area	0.06	1.81	23015	<2E-16
Climate Isolation	0.06	1.25	13158	<2E-16
Bio1	0.11	1.37	42469	<2E-16
Bio12	0.02	2.14	6348	<2E-16
Joint Contribution within the geography of climate	0.06			
Joint Contribution within climate itself	0.01			
Joint Contribution between the geography of climate and climate itself	0.60			
Total	0.92			
**Birds (40 equal interval resolution)** - *Adjusted R^2 = 0.90, Predicted R^2 = 0.89*				
Climate Area	0.07	1.60	28355	<2E-16
Climate Isolation	0.06	1.23	15187	<2E-16
Bio1	0.12	1.37	56011	<2E-16

Bio12	0.02	1.81	9219	<2E-16
Joint Contribution within the geography of climate	0.06			
Joint Contribution within climate itself	0.02			
Joint Contribution between the geography of climate and climate itself	0.58			
Total	0.92			
**Birds (50 equal interval resolution)** - *Adjusted R^2 = 0.90, Predicted R^2 = 0.89*				
Climate Area	0.07	1.49	36518	<2E-16
Climate Isolation	0.07	1.22	17896	<2E-16
Bio1	0.14	1.27	86143	<2E-16
Bio12	0.02	1.67	9280	<2E-16
Joint Contribution within the geography of climate	0.09			
Joint Contribution within climate itself	0.01			
Joint Contribution between the geography of climate and climate itself	0.51			
Total	0.90			
**Birds (60 equal interval resolution)** - *Adjusted R^2 = 0.88, Predicted R^2 = 0.87*				
Climate Area	0.07	1.39	39406	<2E-16
Climate Isolation	0.06	1.20	21974	<2E-16
Bio1	0.16	1.21	104296	<2E-16
Bio12	0.02	1.53	11137	<2E-16
Joint Contribution within the geography of climate	0.09			
Joint Contribution within climate itself	0.02			
Joint Contribution between the geography of climate and climate itself	0.46			
Total	0.86			

Supplementary Table 11 | Observed statistics of general additive models for mammals when models are fitted assuming different resolutions of climate space. Models were fitted assuming the richness patterns of mammals as response variable and climate area, climate isolation, **mean annual temperature** and **mean annual precipitation** as predictors. Here climate space is defined by ***temperature (Bio1)*** and ***precipitation (Bio12)*** as explained in *Sensitivity analysis for different definitions of the climate space* section. The gridded climate space is defined based on 20, 30, 40, 50 and 60 equal interval divisions of the climate axis. As with Supplementary Table 1, each predictor's importance is assessed by the reduction in deviance when it is excluded from the full model. The p-values for each variable are computed using two-sided tests.

	Proportion of Null Deviance	VIF	Chi.sq	p-value
Mammals (20 equal interval resolution) *- Adjusted R^2 = 0.93, Predicted R^2 = 0.93*				
Climate Area	0.06	2.14	4358	<2E-16
Climate Isolation	0.06	1.35	2147	<2E-16
Bio1	0.11	1.46	10718	<2E-16
Bio12	0.03	2.62	2992	<2E-16
Joint Contribution within the geography of climate	0.06			
Joint Contribution within climate itself	0.02			
Joint Contribution between the geography of climate and climate itself	0.58			
Total	0.92			
Mammals (30 equal interval resolution) *- Adjusted R^2 = 0.91, Predicted R^2 = 0.91*				
Climate Area	0.07	1.81	9871	<2E-16
Climate Isolation	0.06	1.25	3909	<2E-16
Bio1	0.10	1.35	12838	<2E-16
Bio12	0.02	2.13	2544	<2E-16
Joint Contribution within the geography of climate	0.06			
Joint Contribution within climate itself	0.00			
Joint Contribution between the geography of climate and climate itself	0.60			
Total	0.91			
**Mammals (40 equal interval resolution)** *- Adjusted R^2 = 0.90, Predicted R^2 = 0.89*				
Climate Area	0.08	1.60	11448	<2E-16
Climate Isolation	0.05	1.23	4491	<2E-16
Bio1	0.11	1.32	18111	<2E-16

Bio12	0.02	1.81	4045	<2E-16
Joint Contribution within the geography of climate	0.06			
Joint Contribution within climate itself	0.02			
Joint Contribution between the geography of climate and climate itself	0.57			
Total	0.89			
**Mammals (50 equal interval resolution)** - *Adjusted R^2 = 0.89, Predicted R^2 = 0.88*				
Climate Area	0.07	1.49	14450	<2E-16
Climate Isolation	0.06	1.22	5399	<2E-16
Bio1	0.12	1.27	25691	<2E-16
Bio12	0.02	1.67	3731	<2E-16
Joint Contribution within the geography of climate	0.09			
Joint Contribution within climate itself	0.00			
Joint Contribution between the geography of climate and climate itself	0.50			
Total	0.88			
**Mammals (60 equal interval resolution)** - *Adjusted R^2 = 0.87, Predicted R^2 = 0.86*				
Climate Area	0.06	1.38	15854	<2E-16
Climate Isolation	0.06	1.20	6876	<2E-16
Bio1	0.15	1.21	31644	<2E-16
Bio12	0.02	1.52	3970	<2E-16
Joint Contribution within the geography of climate	0.10			
Joint Contribution within climate itself	0.02			
Joint Contribution between the geography of climate and climate itself	0.45			
Total	0.86			

Supplementary Table 12 | Observed statistics of general additive models for amphibians when models are fitted assuming different resolutions of climate space. Models were fitted assuming the richness patterns of amphibians as response variable and climate area, climate isolation, **mean annual temperature** and **mean annual precipitation** as predictors. Here climate space is defined by ***temperature (Bio1)*** and ***precipitation (Bio12)*** as explained in *Sensitivity analysis for different definitions of the climate space* section. The gridded climate space is defined based on 20, 30, 40, 50 and 60 equal interval divisions of the climate axis. As with Supplementary Table 1, each predictor's importance is assessed by the reduction in deviance when it is excluded from the full model. The p-values for each variable are computed using two-sided tests.

	Proportion of Null Deviance	VIF	Chi.sq	p-value
Amphibians (20 equal interval resolution) *- Adjusted R^2 = 0.91, Predicted R^2 = 0.91*				
Climate Area	0.06	2.06	2847	<2E-16
Climate Isolation	0.05	1.14	1708	<2E-16
Bio1	0.13	1.40	8081	<2E-16
Bio12	0.02	2.43	1285	<2E-16
Joint Contribution within the geography of climate	0.08			
Joint Contribution within climate itself	0.00			
Joint Contribution between the geography of climate and climate itself	0.57			
Total	0.90			
Amphibians (30 equal interval resolution) *- Adjusted R^2 = 0.89, Predicted R^2 = 0.89*				
Climate Area	0.08	1.77	7396	<2E-16
Climate Isolation	0.06	1.13	2812	<2E-16
Bio1	0.12	1.30	9797	<2E-16
Bio12	0.01	2.05	1266	<2E-16
Joint Contribution within the geography of climate	0.06			
Joint Contribution within climate itself	0.01			
Joint Contribution between the geography of climate and climate itself	0.54			
Total	0.88			
**Amphibians (40 equal interval resolution)** *- Adjusted R^2 = 0.86, Predicted R^2 = 0.84*				
Climate Area	0.06	1.56	6117	<2E-16
Climate Isolation	0.05	1.14	2819	<2E-16
Bio1	0.13	1.31	13099	<2E-16

Bio12	0.01	1.75	1030	<2E-16
Joint Contribution within the geography of climate	0.06			
Joint Contribution within climate itself	0.00			
Joint Contribution between the geography of climate and climate itself	0.53			
Total	0.86			
**Amphibians (50 equal interval resolution)** *- Adjusted R^2 = 0.85, Predicted R^2 = 0.83*				
Climate Area	0.07	1.46	7901	<2E-16
Climate Isolation	0.06	1.11	2593	<2E-16
Bio1	0.16	1.26	19449	<2E-16
Bio12	0.01	1.63	1147	<2E-16
Joint Contribution within the geography of climate	0.09			
Joint Contribution within climate itself	0.01			
Joint Contribution between the geography of climate and climate itself	0.44			
Total	0.84			
**Amphibians (60 equal interval resolution)** *- Adjusted R^2 = 0.82, Predicted R^2 = 0.80*				
Climate Area	0.05	1.37	7837	<2E-16
Climate Isolation	0.06	1.10	3672	<2E-16
Bio1	0.19	1.20	20467	<2E-16
Bio12	0.01	1.50	868	<2E-16
Joint Contribution within the geography of climate	0.09			
Joint Contribution within climate itself	0.01			
Joint Contribution between the geography of climate and climate itself	0.40			
Total	0.81			

Supplementary Table 13 | Observed statistics of general additive models for reptiles when models are fitted assuming different resolutions of climate space. Models were fitted assuming the richness patterns of reptiles as response variable and climate area, climate isolation, **mean annual temperature** and **mean annual precipitation** as predictors. Here climate space is defined by ***temperature (Bio1)*** and ***precipitation (Bio12)*** as explained in *Sensitivity analysis for different definitions of the climate space* section. The gridded climate space is defined based on 20, 30, 40, 50 and 60 equal interval divisions of the climate axis. As with Supplementary Table 1, each predictor's importance is assessed by the reduction in deviance when it is excluded from the full model. The p-values for each variable are computed using two-sided tests.

Reptiles (20 equal interval resolution) *- Adjusted R^2 = 0.94, Predicted R^2 = 0.94*				
	**Proportion of Null Deviance**	**VIF**	**Chi.sq**	**p-value**
Climate Area	0.05	2.06	5419	<2E-16
Climate Isolation	0.05	1.13	2882	<2E-16
Bio1	0.15	1.40	17729	<2E-16
Bio12	0.03	2.49	2771	<2E-16
Joint Contribution within the geography of climate	0.06			
Joint Contribution within climate itself	0.01			
Joint Contribution between the geography of climate and climate itself	0.58			
Total	0.93			
Reptiles (30 equal interval resolution) *- Adjusted R^2 = 0.92, Predicted R^2 = 0.92*				
Climate Area	0.06	1.75	11520	<2E-16
Climate Isolation	0.04	1.08	4425	<2E-16
Bio1	0.15	1.36	23525	<2E-16
Bio12	0.02	2.09	1741	<2E-16
Joint Contribution within the geography of climate	0.05			
Joint Contribution within climate itself	0.00			
Joint Contribution between the geography of climate and climate itself	0.59			
Total	0.91			
**Reptiles (40 equal interval resolution)** *- Adjusted R^2 = 0.90, Predicted R^2 = 0.89*				
Climate Area	0.05	1.57	11866	<2E-16
Climate Isolation	0.04	1.10	5879	<2E-16
Bio1	0.16	1.31	30472	<2E-16

Bio12	0.02	1.80	2518	<2E-16
Joint Contribution within the geography of climate	0.05			
Joint Contribution within climate itself	0.01			
Joint Contribution between the geography of climate and climate itself	0.57			
Total	0.90			
**Reptiles (50 equal interval resolution)** - *Adjusted R^2 = 0.90, Predicted R^2 = 0.89*				
Climate Area	0.06	1.45	14456	<2E-16
Climate Isolation	0.05	1.07	7303	<2E-16
Bio1	0.19	1.28	42218	<2E-16
Bio12	0.01	1.68	1748	<2E-16
Joint Contribution within the geography of climate	0.08			
Joint Contribution within climate itself	0.00			
Joint Contribution between the geography of climate and climate itself	0.5			
Total	0.89			
**Reptiles (60 equal interval resolution)** - *Adjusted R^2 = 0.89, Predicted R^2 = 0.87*				
Climate Area	0.05	1.35	14911	<2E-16
Climate Isolation	0.05	1.07	9070	<2E-16
Bio1	0.21	1.23	50429	<2E-16
Bio12	0.02	1.54	2187	<2E-16
Joint Contribution within the geography of climate	0.08			
Joint Contribution within climate itself	0.00			
Joint Contribution between the geography of climate and climate itself	0.46			
Total	0.87			

Supplementary Table 14 | Observed statistics of general additive models for birds when models are fitted assuming different resolutions of climate space. Models were fitted assuming the richness patterns of birds as response variable and climate area, climate isolation and principal components one and two as predictors. Here climate space is defined by the first two axes of a PCA with the twelve climate variables used in the main text, but prior to the PCA, **precipitation variables were log transformed**. The gridded climate space is defined based on 20, 30, 40, 50 and 60 equal interval divisions of the climate axis. As with Supplementary Table 1, each predictor's importance is assessed by the reduction in deviance when it is excluded from the full model. The p-values for each variable are computed using two-sided tests.

Birds (20 equal interval resolution) *- Adjusted R^2 = 0.92, Predicted R^2 = 0.92*				
	**Proportion of Null Deviance**	**VIF**	**Chi.sq**	**p-value**
Climate Area	0.07	1.12	15778	<2E-16
Climate Isolation	0.05	1.73	10001	<2E-16
PC1	0.11	1.41	34638	<2E-16
PC2	0.02	1.19	6228	<2E-16
Joint Contribution within the geography of climate	0.03			
Joint Contribution within climate itself	0.02			
Joint Contribution between the geography of climate and climate itself	0.62			
Total	0.92			
Birds (30 equal interval resolution) *- Adjusted R^2 = 0.90, Predicted R^2 = 0.91*				
Climate Area	0.05	1.10	21083	<2E-16
Climate Isolation	0.05	1.45	18109	<2E-16
PC1	0.13	1.27	61687	<2E-16
PC2	0.04	1.10	15955	<2E-16
Joint Contribution within the geography of climate	0.07			
Joint Contribution within climate itself	0.04			
Joint Contribution between the geography of climate and climate itself	0.52			
Total	0.90			
**Birds (40 equal interval resolution)** *- Adjusted R^2 = 0.90, Predicted R^2 =0.91*				
Climate Area	0.08	1.06	38541	<2E-16
Climate Isolation	0.04	1.35	19354	<2E-16
PC1	0.14	1.23	89190	<2E-16
PC2	0.04	1.07	21811	<2E-16

Joint Contribution within the geography of climate	0.05			
Joint Contribution within climate itself	0.03			
Joint Contribution between the geography of climate and climate itself	0.52			
Total	0.90			
**Birds (50 equal interval resolution)** - *Adjusted R^2 = 0.89, Predicted R^2 = 0.90*				
Climate Area	0.07	1.06	49079	<2E-16
Climate Isolation	0.04	1.30	23609	<2E-16
PC1	0.15	1.19	115160	<2E-16
PC2	0.05	1.06	31028	<2E-16
Joint Contribution within the geography of climate	0.07			
Joint Contribution within climate itself	0.03			
Joint Contribution between the geography of climate and climate itself	0.47			
Total	0.88			
**Birds (60 equal interval resolution)** - *Adjusted R^2 = 0.87, Predicted R^2 = 0.90*				
Climate Area	0.07	1.07	50475	<2E-16
Climate Isolation	0.04	1.24	24324	<2E-16
PC1	0.17	1.15	154386	<2E-16
PC2	0.05	1.05	42355	<2E-16
Joint Contribution within the geography of climate	0.10			
Joint Contribution within climate itself	0.04			
Joint Contribution between the geography of climate and climate itself	0.40			
Total	0.87			

Supplementary Table 15 | Observed statistics of general additive models for mammals when models are fitted assuming different resolutions of climate space. Models were fitted assuming the richness patterns of mammals as response variable and climate area, climate isolation and principal components one and two as predictors. Here climate space is defined by the first two axes of a PCA with the twelve climate variables used in the main text, but prior to the PCA, **precipitation variables were log transformed**. The gridded climate space is defined based on 20, 30, 40, 50 and 60 equal interval divisions of the climate axis. As with Supplementary Table 1, each predictor's importance is assessed by the reduction in deviance when it is excluded from the full model. The p-values for each variable are computed using two-sided tests.

	Proportion of Null Deviance	VIF	Chi.sq	p-value
Mammals (20 equal interval resolution) *- Adjusted R^2 = 0.91, Predicted R^2 = 0.92*				
Climate Area	0.08	1.11	6571	<2E-16
Climate Isolation	0.04	1.72	2348	<2E-16
PC1	0.11	1.41	12125	<2E-16
PC2	0.04	1.19	3409	<2E-16
Joint Contribution within the geography of climate	0.03			
Joint Contribution within climate itself	0.04			
Joint Contribution between the geography of climate and climate itself	0.57			
Total	0.90			
Mammals (30 equal interval resolution) *- Adjusted R^2 = 0.89, Predicted R^2 = 0.90*				
Climate Area	0.06	1.10	8625	<2E-16
Climate Isolation	0.04	1.48	4234	<2E-16
PC1	0.13	1.29	20280	<2E-16
PC2	0.05	1.11	7265	<2E-16
Joint Contribution within the geography of climate	0.07			
Joint Contribution within climate itself	0.05			
Joint Contribution between the geography of climate and climate itself	0.49			
Total	0.89			
***Mammals* (40 equal interval resolution)** *- Adjusted R^2 = 0.88, Predicted R^2 = 0.90*				
Climate Area	0.08	1.06	15451	<2E-16
Climate Isolation	0.03	1.35	4494	<2E-16
PC1	0.14	1.24	28275	<2E-16
PC2	0.05	1.07	9080	<2E-16

Joint Contribution within the geography of climate	0.05			
Joint Contribution within climate itself	0.04			
Joint Contribution between the geography of climate and climate itself	0.49			
Total	0.88			
Mammals **(50 equal interval resolution)** *- Adjusted R^2 = 0.87, Predicted R^2 =0.88*				
Climate Area	0.08	1.06	18934	<2E-16
Climate Isolation	0.03	1.31	5420	<2E-16
PC1	0.15	1.20	37638	<2E-16
PC2	0.06	1.07	12320	<2E-16
Joint Contribution within the geography of climate	0.07			
Joint Contribution within climate itself	0.05			
Joint Contribution between the geography of climate and climate itself	0.43			
Total	0.87			
Mammals **(60 equal interval resolution)** *- Adjusted R^2 = 0.86, Predicted R^2 =0.88*				
Climate Area	0.07	1.06	19090	<2E-16
Climate Isolation	0.03	1.24	5551	<2E-16
PC1	0.17	1.15	49347	<2E-16
PC2	0.06	1.05	15917	<2E-16
Joint Contribution within the geography of climate	0.11			
Joint Contribution within climate itself	0.04			
Joint Contribution between the geography of climate and climate itself	0.37			
Total	0.85			

Supplementary Table 16 | Observed statistics of general additive models for amphibians when models are fitted assuming different resolutions of climate space. Models were fitted assuming the richness patterns of amphibians as response variable and climate area, climate isolation and principal components one and two as predictors. Here climate space is defined by the first two axes of a PCA with the twelve climate variables used in the main text, but prior to the PCA, **precipitation variables were log transformed**. The gridded climate space is defined based on 20, 30, 40, 50 and 60 equal interval divisions of the climate axis. As with Supplementary Table 1, each predictor's importance is assessed by the reduction in deviance when it is excluded from the full model. The p-values for each variable are computed using two-sided tests.

	Proportion of Null Deviance	VIF	Chi.sq	p-value
***Amphibians (20 equal interval resolution)** - Adjusted R^2 = 0.90, Predicted R^2 = 0.90*				
Climate Area	0.06	1.08	4450	<2E-16
Climate Isolation	0.05	1.58	2615	<2E-16
PC1	0.13	1.32	10369	<2E-16
PC2	0.02	1.18	1074	<2E-16
Joint Contribution within the geography of climate	0.02			
Joint Contribution within climate itself	0.01			
Joint Contribution between the geography of climate and climate itself	0.60			
Total	0.89			
***Amphibians (30 equal interval resolution)** - Adjusted R^2 = 0.85, Predicted R^2 = 0.87*				
Climate Area	0.04	1.05	4690	<2E-16
Climate Isolation	0.04	1.45	2862	<2E-16
PC1	0.15	1.27	16443	<2E-16
PC2	0.03	1.13	2852	<2E-16
Joint Contribution within the geography of climate	0.07			
Joint Contribution within climate itself	0.03			
Joint Contribution between the geography of climate and climate itself	0.49			
Total	0.85			
***Amphibians** **(40 equal interval resolution)** - Adjusted R^2 = 0.85, Predicted R^2 = 0.87*				
Climate Area	0.05	1.02	7260	<2E-16
Climate Isolation	0.04	1.28	3879	<2E-16
PC1	0.16	1.18	20177	<2E-16
PC2	0.03	1.07	3247	<2E-16

Joint Contribution within the geography of climate	0.05			
Joint Contribution within climate itself	0.01			
Joint Contribution between the geography of climate and climate itself	0.50			
Total	0.84			
***Amphibians*(50 equal interval resolution)** - *Adjusted R^2 = 0.83, Predicted R^2 = 0.85*				
Climate Area	0.05	1.03	8911	<2E-16
Climate Isolation	0.03	1.23	4172	<2E-16
PC1	0.18	1.15	25130	<2E-16
PC2	0.04	1.06	4354	<2E-16
Joint Contribution within the geography of climate	0.06			
Joint Contribution within climate itself	0.02			
Joint Contribution between the geography of climate and climate itself	0.44			
Total	0.82			
***Amphibians* (60 equal interval resolution)** - *Adjusted R^2 = 0.80, Predicted R^2 = 0.84*				
Climate Area	0.04	1.03	8074	<2E-16
Climate Isolation	0.03	1.17	3600	<2E-16
PC1	0.21	1.11	31497	<2E-16
PC2	0.05	1.03	6306	<2E-16
Joint Contribution within the geography of climate	0.08			
Joint Contribution within climate itself	0.03			
Joint Contribution between the geography of climate and climate itself	0.36			
Total	0.80			

Supplementary Table 17 | Observed statistics of general additive models for reptiles when models are fitted assuming different resolutions of climate space. Models were fitted assuming the richness patterns of reptiles as response variable and climate area, climate isolation and principal components one and two as predictors. Here climate space is defined by the first two axes of a PCA with the twelve climate variables used in the main text, but prior to the PCA, **precipitation variables were log transformed**. The gridded climate space is defined based on 20, 30, 40, 50 and 60 equal interval divisions of the climate axis. As with Supplementary Table 1, each predictor's importance is assessed by the reduction in deviance when it is excluded from the full model.

Reptiles (20 equal interval resolution) - *Adjusted R^2 = 0.92 , Predicted R^2 = 0.92*				
	**Proportion of Null Deviance**	**VIF**	**Chi.sq**	**p-value**
Climate Area	0.08	1.08	7814	<2E-16
Climate Isolation	0.05	1.65	5355	<2E-16
PC1	0.14	1.28	19515	<2E-16
PC2	0.01	1.25	1474	<2E-16
Joint Contribution within the geography of climate	0.02			
Joint Contribution within climate itself	0.01			
Joint Contribution between the geography of climate and climate itself	0.61			
Total	0.91			
Reptiles (30 equal interval resolution) - *Adjusted R^2 = 0.89, Predicted R^2 = 0.89*				
Climate Area	0.05	1.07	10061	<2E-16
Climate Isolation	0.04	1.41	7592	<2E-16
PC1	0.17	1.17	31236	<2E-16
PC2	0.02	1.16	3877	<2E-16
Joint Contribution within the geography of climate	0.06			
Joint Contribution within climate itself	0.00			
Joint Contribution between the geography of climate and climate itself	0.54			
Total	0.88			
***Reptiles* (40 equal interval resolution)** - *Adjusted R^2 = 0.89, Predicted R^2 = 0.89*				
Climate Area	0.06	1.03	15017	<2E-16
Climate Isolation	0.04	1.32	9581	<2E-16
PC1	0.20	1.15	44259	<2E-16
PC2	0.02	1.13	4588	<2E-16

Joint Contribution within the geography of climate	0.04			
Joint Contribution within climate itself	0.00			
Joint Contribution between the geography of climate and climate itself	0.52			
Total	0.87			
***Reptiles* (50 equal interval resolution)** - *Adjusted R^2 = 0.88, Predicted R^2 = 0.88*				
Climate Area	0.06	1.04	18442	<2E-16
Climate Isolation	0.04	1.22	11230	<2E-16
PC1	0.23	1.12	55451	<2E-16
PC2	0.02	1.11	6398	<2E-16
Joint Contribution within the geography of climate	0.05			
Joint Contribution within climate itself	0.00			
Joint Contribution between the geography of climate and climate itself	0.47			
Total	0.87			
***Reptiles* (60 equal interval resolution)** - *Adjusted R^2 = 0.87, Predicted R^2 = 0.88*				
Climate Area	0.06	1.05	19521	<2E-16
Climate Isolation	0.03	1.22	10581	<2E-16
PC1	0.26	1.07	66239	<2E-16
PC2	0.02	1.10	8868	<2E-16
Joint Contribution within the geography of climate	0.07			
Joint Contribution within climate itself	0.01			
Joint Contribution between the geography of climate and climate itself	0.41			
Total	0.86			

Supplementary Table 18. Comparison of Deviance Reduction for Linear and Polynomial Generalized Linear Models. This table presents the results of an Analysis of Variance (ANOVA) test performed on a set of Generalized Linear Models (GLMs) with varying degrees of polynomial terms, as well as the gain in explanatory power with different degrees of non-linear flexibility. The GLMs range from a linear model (no polynomial terms) to polynomial models of degrees two through five. The purpose of this ANOVA is to compare the goodness-of-fit of these models to the data, as indicated by the reduction in deviance from one model to the next. The deviance, a measure of the discrepancy between the observed data and the model's predictions, is given for each model, showing how well each model fits the data. Lower deviance values indicate a better fit. Please note that the linear model does not have a deviance value as it serves as the reference model for comparison with the polynomial models. Column "McFadden's R2" indicates the goodness-of-fit of each GLM as measured by McFadden's pseudo R2, and column "Delta R2" indicates the gain in goodness-of-fit by the non-linear models when compared to the linear model.

Models	Residual Deviance	Degrees of Freedom	Deviance	McFadden's R^2	Delta R^2 (Polynomial - Linear)
**Birds**					
Linear	54674			0.758	
Polynomial 2nd degree	24799	4	29875.1	0.879	0.121
Polynomial 3rd degree	20791	4	4008.1	0.898	0.140
Polynomial 4th degree	19852	4	939.5	0.902	0.144
Polynomial 5th degree	18337	4	1514.4	0.909	0.151
**Mammals**					
Linear	20489.3				
Polynomial 2nd degree	9003.9	4	11485.4	0.86	0.13
Polynomial 3rd degree	7542.9	4	1461	0.88	0.15
Polynomial 4th degree	7090.4	4	452.4	0.89	0.16
Polynomial 5th degree	6537.4	4	553.1	0.90	0.17
**Amphibians**					
Linear	18756.1				
Polynomial 2nd degree	9343.5	4	9412.7	0.83	0.12
Polynomial 3rd degree	7944.1	4	1399.4	0.85	0.14

Polynomial 4th degree	7660.8	4	283.3	0.86	0.15
Polynomial 5th degree	7164.9	4	495.9	0.87	0.16
**Reptiles**					
Linear	28386.1				
Polynomial 2nd degree	13036.1	4	15349.9	0.86	0.1
Polynomial 3rd degree	11186.9	4	1849.2	0.88	0.12
Polynomial 4th degree	9700	4	543.7	0.89	0.13
Polynomial 5th degree	9156.3	4	1486.9	0.90	0.14

Supplementary Table 19 | Degree-4 Polynomial Regression Outcomes for Bird Richness in Relation to Geography of Climate and Climate Itself, Assessed Across Various Climate Space Resolutions. This table showcases the impact of geography of climate (climate area and climate isolation) and climate itself (principal components one and two) on bird richness, as determined by a degree-4 polynomial regression. The analysis was carried out using different grids of climate spaces, divided into 20, 30, 40, 50, and 60 equal intervals along the climate axis. The approach of deviance reduction, as outlined in Supplementary Table 1, was applied here to assess the importance of each predictor. The p-values for each variable are computed using two-sided tests.

Birds (20 equal intervals)

	Estimate	Std. Error	z value	Pr(>|z|)
Intercept	6.98	0.00	2417.84	< 0.001
Climate Area1	5.16	0.04	129.15	< 0.001
Climate Area2	-2.26	0.03	-72.60	< 0.001
Climate Area3	1.37	0.03	50.79	< 0.001
Climate Area4	-0.65	0.02	-26.94	< 0.001
Climate Isolation1	4.65	0.06	80.84	< 0.001
Climate Isolation2	-2.13	0.04	-51.67	< 0.001
Climate Isolation3	-0.27	0.04	-6.57	< 0.001
Climate Isolation4	-0.37	0.03	-11.27	< 0.001
PC1_1	7.46	0.06	131.80	< 0.001
PC1_2	-1.10	0.06	-18.48	< 0.001
PC1_3	-1.27	0.04	-30.67	< 0.001
PC1_4	0.40	0.04	9.30	< 0.001
PC2_1	-0.38	0.07	-5.63	< 0.001
PC2_2	-3.93	0.06	-67.13	< 0.001
PC2_3	-0.77	0.05	-15.76	< 0.001
PC2_4	-0.35	0.04	-8.03	< 0.001

**Predictor's Importance**		**Model Goodness-of-fit**	
**Predictor Importance**	**Proportion of Null Deviance**	*Null Deviance*	216861.00
Climate Area	0.09	*Residual Deviance*	19852.00
Climate Isolation	0.04	*AIC*	21321.00
PC1	0.14	*McFadden's R^2*	0.90
PC2	0.03	Predicted R^2	0.93
Joint Contribution within the geography of climate	0.11		

Joint Contribution within climate itself	0.00
Joint Contribution between the geography of climate and climate itself	0.50
Total	0.91

Birds (30 equal intervals)

	Estimate	Std. Error	z value	Pr(>|z|)
Intercept	6.74	0.00	2892.05	< 0.001
Climate Area1	6.83	0.04	163.57	< 0.001
Climate Area2	-3.33	0.03	-102.60	< 0.001
Climate Area3	2.00	0.03	74.38	< 0.001
Climate Area4	-1.18	0.02	-50.18	< 0.001
Climate Isolation1	7.77	0.06	125.73	< 0.001
Climate Isolation2	-2.71	0.05	-56.82	< 0.001
Climate Isolation3	0.51	0.04	12.83	< 0.001
Climate Isolation4	-0.36	0.04	-9.71	< 0.001
PC1_1	10.89	0.06	173.24	< 0.001
PC1_2	-0.32	0.07	-4.85	< 0.001
PC1_3	-1.48	0.05	-30.34	< 0.001
PC1_4	1.11	0.05	22.02	< 0.001
PC2_1	-0.52	0.07	-7.18	< 0.001
PC2_2	-5.06	0.07	-72.34	< 0.001
PC2_3	-1.34	0.05	-25.14	< 0.001
PC2_4	0.71	0.05	15.09	< 0.001

Predictor's Importance		Model Goodness-of-fit	
**Predictor Importance**	**Proportion of Null Deviance**	*Null Deviance*	357218.00
Climate Area	0.08	*Residual Deviance*	31483.00
Climate Isolation	0.05	*AIC*	34294.00
PC1	0.15	*McFadden's R2*	0.90
PC2	0.03	Predicted R^2	0.92
Joint Contribution within the geography of climate	0.11		
Joint Contribution within climate itself	0.02		

Joint Contribution between the geography of climate and climate itself	0.47		
Total	0.91		

Birds (40 equal intervals)

	Estimate	Std. Error	z value	Pr(>|z|)
Intercept	6.64	0.00	3533.35	< 0.001
Climate Area1	8.22	0.04	194.09	< 0.001
Climate Area2	-3.58	0.03	-107.23	< 0.001
Climate Area3	2.04	0.03	72.25	< 0.001
Climate Area4	-1.24	0.02	-50.30	< 0.001
Climate Isolation1	7.41	0.06	125.30	< 0.001
Climate Isolation2	-2.21	0.05	-44.70	< 0.001
Climate Isolation3	-0.71	0.04	-16.20	< 0.001
Climate Isolation4	0.11	0.04	2.60	0.01
PC1_1	13.47	0.06	211.35	< 0.001
PC1_2	-1.32	0.06	-20.91	< 0.001
PC1_3	-0.92	0.05	-18.29	< 0.001
PC1_4	0.74	0.05	14.83	< 0.001
PC2_1	-1.39	0.07	-19.83	< 0.001
PC2_2	-6.79	0.07	-96.66	< 0.001
PC2_3	-1.32	0.05	-24.64	< 0.001
PC2_4	0.08	0.05	1.60	0.11

Predictor's Importance		Model Goodness-of-fit	
**Predictor Importance**	**Proportion of Null Deviance**	*Null Deviance*	459470.00
Climate Area	0.09	*Residual Deviance*	43044.00
Climate Isolation	0.04	*AIC*	47496.00
PC1	0.15	*McFadden's R2*	0.90
PC2	0.03	Predicted R^2	0.92
Joint Contribution within the geography of climate	0.08		
Joint Contribution within climate itself	0.02		

Joint Contribution between the geography of climate and climate itself	0.50			
Total	0.91			

Birds (50 equal intervals)

	Estimate	Std. Error	z value	Pr(>|z|)
Intercept	6.54	0.00	3985.33	< 0.001
Climate Area1	9.55	0.04	217.32	< 0.001
Climate Area2	-4.42	0.04	-120.83	< 0.001
Climate Area3	2.48	0.03	79.69	< 0.001
Climate Area4	-1.32	0.03	-49.77	< 0.001
Climate Isolation1	7.03	0.06	119.25	< 0.001
Climate Isolation2	-3.03	0.05	-63.24	< 0.001
Climate Isolation3	-0.30	0.04	-7.27	< 0.001
Climate Isolation4	0.31	0.04	7.90	< 0.001
PC1_1	16.37	0.06	254.59	< 0.001
PC1_2	-2.26	0.06	-34.91	< 0.001
PC1_3	-1.18	0.05	-21.74	< 0.001
PC1_4	0.53	0.05	9.99	< 0.001
PC2_1	-3.84	0.07	-53.81	< 0.001
PC2_2	-7.50	0.07	-102.65	< 0.001
PC2_3	-2.16	0.06	-38.53	< 0.001
PC2_4	0.05	0.05	1.03	0.30

**Predictor's Importance**		**Model Goodness-of-fit**	
**Predictor Importance**	**Proportion of Null Deviance**	*Null Deviance*	556414.00
Climate Area	0.09	*Residual Deviance*	59992.00
Climate Isolation	0.03	*AIC*	66308.00
PC1	0.18	*McFadden's R2*	0.88
PC2	0.04	Predicted R^2	0.90
Joint Contribution within the geography of climate	0.08		
Joint Contribution within climate itself	0.01		

Joint Contribution between the geography of climate and climate itself	0.46
Total	0.89

Birds (60 equal intervals)

	Estimate	Std. Error	z value	Pr(>|z|)
Intercept	6.46	0.00	4449.24	< 0.001
Climate Area1	10.75	0.05	234.17	< 0.001
Climate Area2	-4.80	0.04	-125.24	< 0.001
Climate Area3	2.72	0.03	79.85	< 0.001
Climate Area4	-1.63	0.03	-56.12	< 0.001
Climate Isolation1	7.16	0.06	119.33	< 0.001
Climate Isolation2	-2.83	0.05	-57.64	< 0.001
Climate Isolation3	-0.78	0.04	-18.69	< 0.001
Climate Isolation4	0.00	0.04	-0.06	0.95
PC1_1	18.94	0.07	282.87	< 0.001
PC1_2	-3.18	0.07	-46.95	< 0.001
PC1_3	-1.15	0.06	-20.27	< 0.001
PC1_4	0.50	0.06	8.78	< 0.001
PC2_1	-5.07	0.08	-66.95	< 0.001
PC2_2	-8.76	0.08	-112.66	< 0.001
PC2_3	-2.58	0.06	-42.15	< 0.001
PC2_4	0.40	0.05	7.48	< 0.001

Predictor's Importance		Model Goodness-of-fit	
**Predictor Importance**	**Proportion of Null Deviance**	*Null Deviance*	639705.00
Climate Area	0.09	*Residual Deviance*	73768.00
Climate Isolation	0.03	*AIC*	82256.00
PC1	0.19	*McFadden's R2*	0.87
PC2	0.04	Predicted R^2	0.90
Joint Contribution within the geography of climate	0.09		
Joint Contribution within climate itself	0.02		

Joint Contribution between the geography of climate and climate itself	0.42	
Total	0.88	

Supplementary Table 20 | Degree-4 Polynomial Regression Outcomes for Mammal Richness in Relation to Geography of Climate and Climate Itself, Assessed Across Various Climate Space Resolutions. This table showcases the impact of geography of climate (climate area and climate isolation) and climate itself (principal components one and two) on bird richness, as determined by a degree-4 polynomial regression. The analysis was carried out using different grids of climate spaces, divided into 20, 30, 40, 50, and 60 equal intervals along the climate axis. The approach of deviance reduction, as outlined in Supplementary Table 1, was applied here to assess the importance of each predictor. The p-values for each variable are computed using two-sided tests.

Mammals (20 equal intervals)

	Estimate	Std. Error	z value	Pr(>|z|)
Intercept	5.80	0.01	1086.97	< 0.001
Climate Area1	6.00	0.07	82.67	< 0.001
Climate Area2	-2.84	0.06	-50.86	< 0.001
Climate Area3	1.76	0.05	36.66	< 0.001
Climate Area4	-0.80	0.04	-19.03	< 0.001
Climate Isolation1	4.42	0.10	42.27	< 0.001
Climate Isolation2	-2.19	0.08	-28.27	< 0.001
Climate Isolation3	-0.25	0.07	-3.31	< 0.001
Climate Isolation4	-0.39	0.06	-6.52	< 0.001
PC1_1	7.94	0.10	76.22	< 0.001
PC1_2	-1.55	0.11	-13.81	< 0.001
PC1_3	-0.53	0.08	-6.89	< 0.001
PC1_4	0.15	0.08	1.96	0.05
PC2_1	-0.66	0.12	-5.29	< 0.001
PC2_2	-4.58	0.11	-40.67	< 0.001
PC2_3	-1.14	0.09	-12.41	< 0.001
PC2_4	-0.45	0.08	-5.51	< 0.001

**Predictor's Importance**		**Model Goodness-of-fit**	
**Predictor Importance**	**Proportion of Null Deviance**	*Null Deviance*	76878.30
Climate Area	0.10	*Residual Deviance*	7090.40
Climate Isolation	0.04	*AIC*	8363.00
PC1	0.12	*McFadden's R2*	0.89
PC2	0.03	Predicted R^2	0.93
Joint Contribution within the geography of climate	0.11		
Joint Contribution within climate itself	0.02		

Joint Contribution between the geography of climate and climate itself	0.49
Total	0.91

Mammals (30 equal intervals)

	Estimate	Std. Error	z value	Pr(>|z|)
Intercept	5.54	0.00	1271.85	< 0.001
Climate Area1	7.99	0.08	104.76	< 0.001
Climate Area2	-4.17	0.06	-70.75	< 0.001
Climate Area3	2.59	0.05	53.39	< 0.001
Climate Area4	-1.51	0.04	-36.09	< 0.001
Climate Isolation1	7.11	0.11	62.88	< 0.001
Climate Isolation2	-2.63	0.09	-30.30	< 0.001
Climate Isolation3	0.60	0.07	8.30	< 0.001
Climate Isolation4	-0.42	0.07	-6.12	< 0.001
PC1_1	11.82	0.12	102.30	< 0.001
PC1_2	-0.71	0.12	-5.74	< 0.001
PC1_3	-0.37	0.09	-4.08	< 0.001
PC1_4	0.79	0.09	8.50	< 0.001
PC2_1	-1.10	0.14	-8.08	< 0.001
PC2_2	-6.27	0.13	-46.43	< 0.001
PC2_3	-1.79	0.10	-17.47	< 0.001
PC2_4	0.39	0.09	4.33	< 0.001

Predictor's Importance		Model Goodness-of-fit	
**Predictor Importance**	**Proportion of Null Deviance**	*Null Deviance*	120744.00
Climate Area	0.10	*Residual Deviance*	12088.00
Climate Isolation	0.04	*AIC*	14490.00
PC1	0.14	*McFadden's R2*	0.88
PC2	0.03	Predicted R^2	0.91
Joint Contribution within the geography of climate	0.12		
Joint Contribution within climate itself	0.03		
Joint Contribution between the geography of climate and climate itself	0.44		
Total	0.90		

Mammals (40 equal intervals)

	Estimate	Std. Error	z value	Pr(>|z|)
Intercept	5.42	0.00	1527.05	< 0.001
Climate Area1	9.68	0.08	124.11	< 0.001
Climate Area2	-4.58	0.06	-74.86	< 0.001
Climate Area3	2.78	0.05	54.18	< 0.001
Climate Area4	-1.75	0.04	-39.70	< 0.001
Climate Isolation1	6.30	0.11	58.06	< 0.001
Climate Isolation2	-1.92	0.09	-20.64	< 0.001
Climate Isolation3	-0.66	0.08	-8.03	< 0.001
Climate Isolation4	-0.07	0.08	-0.90	0.37
PC1_1	14.80	0.12	124.72	< 0.001
PC1_2	-2.14	0.12	-17.66	< 0.001
PC1_3	0.39	0.10	4.08	< 0.001
PC1_4	0.21	0.09	2.26	0.02
PC2_1	-2.51	0.13	-18.65	< 0.001
PC2_2	-8.29	0.14	-59.92	< 0.001
PC2_3	-2.02	0.11	-19.12	< 0.001
PC2_4	-0.34	0.09	-3.59	< 0.001

Predictor's Importance		Model Goodness-of-fit	
**Predictor Importance**	**Proportion of Null Deviance**	*Null Deviance*	153673
Climate Area	0.11	*Residual Deviance*	16284
Climate Isolation	0.03	*AIC*	20070
PC1	0.15	*McFadden's R2*	0.87
PC2	0.04	Predicted R^2	0.90
Joint Contribution within the geography of climate	0.09		
Joint Contribution within climate itself	0.03		
Joint Contribution between the geography of climate and climate itself	0.46		
Total	0.91		

Mammals (50 equal intervals)

	Estimate	Std. Error	z value	Pr(>|z|)
Intercept	5.31	0.00	1701.82	< 0.001

Climate Area1	11.15	0.08	137.66	< 0.001
Climate Area2	-5.70	0.07	-84.74	< 0.001
Climate Area3	3.45	0.06	60.80	< 0.001
Climate Area4	-1.91	0.05	-39.66	< 0.001
Climate Isolation1	5.94	0.11	54.62	< 0.001
Climate Isolation2	-2.89	0.09	-32.14	< 0.001
Climate Isolation3	-0.35	0.08	-4.48	< 0.001
Climate Isolation4	0.62	0.07	8.34	< 0.001
PC1_1	17.79	0.12	148.02	< 0.001
PC1_2	-2.75	0.13	-21.84	< 0.001
PC1_3	0.49	0.10	4.78	< 0.001
PC1_4	0.03	0.10	0.31	0.76
PC2_1	-5.07	0.14	-36.90	< 0.001
PC2_2	-9.02	0.14	-62.67	< 0.001
PC2_3	-2.68	0.11	-24.28	< 0.001
PC2_4	-0.49	0.10	-4.90	0.00

**Predictor's Importance**		**Model Goodness-of-fit**	
**Predictor Importance**	**Proportion of Null Deviance**	*Null Deviance*	183905.00
Climate Area	0.11	*Residual Deviance*	20961.00
Climate Isolation	0.03	*AIC*	26309.00
PC1	0.17	*McFadden's R2*	0.86
PC2	0.04	Predicted R^2	0.89
Joint Contribution within the geography of climate	0.09		
Joint Contribution within climate itself	0.02		
Joint Contribution between the geography of climate and climate itself	0.43		
Total	0.89		

Mammals (60 equal intervals)

	**Estimate**	**Std. Error**	**z value**	**Pr(>|z|)**
Intercept	5.22	0.00	1880.84	< 0.001
Climate Area1	12.42	0.08	146.84	< 0.001
Climate Area2	-6.10	0.07	-86.35	< 0.001
Climate Area3	3.72	0.06	59.60	< 0.001

Climate Area4	-2.20	0.05	-41.48	< 0.001
Climate Isolation1	5.82	0.11	52.80	< 0.001
Climate Isolation2	-2.76	0.09	-30.07	< 0.001
Climate Isolation3	-0.64	0.08	-8.27	< 0.001
Climate Isolation4	-0.15	0.07	-2.03	0.04
PC1_1	20.75	0.12	166.74	< 0.001
PC1_2	-3.82	0.13	-29.32	< 0.001
PC1_3	0.54	0.11	5.03	< 0.001
PC1_4	-0.14	0.11	-1.34	0.18
PC2_1	-6.91	0.15	-47.51	< 0.001
PC2_2	-10.46	0.15	-69.61	< 0.001
PC2_3	-3.56	0.12	-29.62	< 0.001
PC2_4	0.07	0.10	0.64	0.52

**Predictor's Importance**		**Model Goodness-of-fit**	
**Predictor Importance**	**Proportion of Null Deviance**	*Null Deviance*	210153.00
Climate Area	0.11	*Residual Deviance*	25984.00
Climate Isolation	0.03	*AIC*	33133.00
PC1	0.19	*McFadden's R2*	0.85
PC2	0.05	Predicted R^2	0.89
Joint Contribution within the geography of climate	0.09		
Joint Contribution within climate itself	0.03		
Joint Contribution between the geography of climate and climate itself	0.38		
Total	0.88		

Supplementary Table 21 | Degree-4 Polynomial Regression Outcomes for Amphibiam Richness in Relation to Geography of Climate and Climate Itself, Assessed Across Various Climate Space Resolutions. This table showcases the impact of geography of climate (climate area and climate isolation) and climate itself (principal components one and two) on bird richness, as determined by a degree-4 polynomial regression. The analysis was carried out using different grids of climate spaces, divided into 20, 30, 40, 50, and 60 equal intervals along the climate axis. The approach of deviance reduction, as outlined in Supplementary Table 1, was applied here to assess the importance of each predictor. The p-values for each variable are computed using two-sided tests.

Amphibians (20 equal intervals)

	Estimate	Std. Error	z value	Pr(>|z|)
Intercept	4.83	0.01	489.26	< 0.001
Climate Area1	7.27	0.10	72.53	< 0.001
Climate Area2	-3.61	0.08	-45.51	< 0.001
Climate Area3	2.13	0.07	30.92	< 0.001
Climate Area4	-0.73	0.06	-12.36	< 0.001
Climate Isolation1	6.22	0.17	36.76	< 0.001
Climate Isolation2	-2.78	0.12	-22.95	< 0.001
Climate Isolation3	-0.21	0.12	-1.70	< 0.001
Climate Isolation4	-0.32	0.09	-3.49	< 0.001
PC1_1	11.02	0.17	63.90	< 0.001
PC1_2	-1.12	0.18	-6.20	< 0.001
PC1_3	-2.43	0.12	-19.76	< 0.001
PC1_4	1.39	0.13	10.74	< 0.001
PC2_1	-2.54	0.22	-11.72	< 0.001
PC2_2	-6.45	0.22	-28.83	< 0.001
PC2_3	-1.97	0.17	-11.26	< 0.001
PC2_4	-1.37	0.15	-9.31	< 0.001

Predictor's Importance		Model Goodness-of-fit	
**Predictor Importance**	**Proportion of Null Deviance**	*Null Deviance*	62080.50
Climate Area	0.11	*Residual Deviance*	7660.80
Climate Isolation	0.03	*AIC*	8704.10
PC1	0.14	*McFadden's R2*	0.86
PC2	0.03	Predicted R^2	0.92
Joint Contribution within the geography of climate	0.10		
Joint Contribution within climate itself	0.00		

Joint Contribution between the geography of climate and climate itself	0.47
Total	0.88

Amphibians (30 equal intervals)

	Estimate	Std. Error	z value	Pr(>|z|)
Intercept	4.58	0.01	580.01	< 0.001
Climate Area1	8.89	0.11	83.90	< 0.001
Climate Area2	-5.01	0.08	-59.75	< 0.001
Climate Area3	3.21	0.07	46.24	< 0.001
Climate Area4	-1.84	0.06	-31.23	< 0.001
Climate Isolation1	7.69	0.18	43.89	< 0.001
Climate Isolation2	-2.55	0.14	-18.88	< 0.001
Climate Isolation3	0.64	0.11	5.98	< 0.001
Climate Isolation4	0.00	0.10	-0.04	< 0.001
PC1_1	16.28	0.19	85.04	< 0.001
PC1_2	0.73	0.20	-3.72	< 0.001
PC1_3	-3.29	0.15	-22.59	< 0.001
PC1_4	2.99	0.15	19.82	< 0.001
PC2_1	-4.36	0.23	-18.67	< 0.001
PC2_2	-8.86	0.26	-34.65	< 0.001
PC2_3	-3.63	0.19	-18.62	< 0.001
PC2_4	-1.02	0.17	-5.92	< 0.001

Predictor's Importance		Model Goodness-of-fit	
**Predictor Importance**	**Proportion of Null Deviance**	*Null Deviance*	81054.00
Climate Area	0.10	*Residual Deviance*	11377.00
Climate Isolation	0.04	*AIC*	13250.00
PC1	0.19	*McFadden's R2*	0.84
PC2	0.03	Predicted R^2	0.89
Joint Contribution within the geography of climate	0.10		
Joint Contribution within climate itself	0.01		
Joint Contribution between the geography of climate and climate itself	0.39		
Total	0.86		

Amphibians (40 equal intervals)

	Estimate	Std. Error	z value	Pr(>|z|)
Intercept	4.36	0.01	618.56	< 0.001
Climate Area1	10.23	0.11	90.59	< 0.001
Climate Area2	-4.93	0.09	-54.76	< 0.001
Climate Area3	3.23	0.08	42.69	< 0.001
Climate Area4	-2.02	0.06	-31.68	< 0.001
Climate Isolation1	7.87	0.17	45.36	< 0.001
Climate Isolation2	-3.01	0.16	-18.68	< 0.001
Climate Isolation3	-1.75	0.14	-12.21	< 0.001
Climate Isolation4	0.46	0.14	3.42	< 0.001
PC1_1	20.00	0.21	94.08	< 0.001
PC1_2	-3.02	0.21	-14.15	< 0.001
PC1_3	-3.06	0.17	-17.78	< 0.001
PC1_4	2.37	0.16	14.79	< 0.001
PC2_1	-6.74	0.24	-28.18	< 0.001
PC2_2	-10.54	0.27	-39.55	< 0.001
PC2_3	-4.89	0.21	-23.75	< 0.001
PC2_4	-2.97	0.18	-16.29	< 0.001

Predictor's Importance		Model Goodness-of-fit	
**Predictor Importance**	**Proportion of Null Deviance**	*Null Deviance*	97439.00
Climate Area	0.10	*Residual Deviance*	13460.00
Climate Isolation	0.03	*AIC*	16357.00
PC1	0.18	*McFadden's R2*	0.84
PC2	0.02	Predicted R^2	0.88
Joint Contribution within the geography of climate	0.08		
Joint Contribution within climate itself	0.01		
Joint Contribution between the geography of climate and climate itself	0.44		
Total	0.86		

Amphibians (50 equal intervals)

	Estimate	Std. Error	z value	Pr(>|z|)
Intercept	4.19	0.01	651.55	< 0.001

Climate Area1	11.51	0.12	94.65	< 0.001
Climate Area2	-6.26	0.10	-61.19	< 0.001
Climate Area3	3.89	0.09	44.99	< 0.001
Climate Area4	-1.94	0.07	-26.94	< 0.001
Climate Isolation1	7.61	0.17	43.72	< 0.001
Climate Isolation2	-4.28	0.15	-28.68	< 0.001
Climate Isolation3	0.21	0.13	1.61	0.11
Climate Isolation4	0.73	0.12	6.17	< 0.001
PC1_1	24.13	0.21	112.87	< 0.001
PC1_2	-3.58	0.21	-16.91	< 0.001
PC1_3	-4.37	0.18	-24.78	< 0.001
PC1_4	4.19	0.18	23.93	< 0.001
PC2_1	-11.74	0.25	-46.13	< 0.001
PC2_2	-12.70	0.29	-43.72	< 0.001
PC2_3	-6.87	0.22	-30.67	< 0.001
PC2_4	-3.75	0.19	-19.40	0.00

**Predictor's Importance**		**Model Goodness-of-fit**	
**Predictor Importance**	**Proportion of Null Deviance**	*Null Deviance*	110213.00
Climate Area	0.09	*Residual Deviance*	17726.00
Climate Isolation	0.02	*AIC*	21712.00
PC1	0.22	*McFadden's R2*	0.81
PC2	0.05	Predicted R^2	0.86
Joint Contribution within the geography of climate	0.08		
Joint Contribution within climate itself	0.00		
Joint Contribution between the geography of climate and climate itself	0.38		
Total	0.84		

Amphibians (60 equal intervals)

	**Estimate**	**Std. Error**	**z value**	**Pr(>|z|)**
Intercept	4.10	0.01	726.38	< 0.001
Climate Area1	11.90	0.13	94.11	< 0.001
Climate Area2	-6.01	0.11	-55.69	< 0.001
Climate Area3	3.89	0.10	40.23	< 0.001

Climate Area4	-2.45	0.08	-30.15	< 0.001
Climate Isolation1	7.50	0.18	42.82	< 0.001
Climate Isolation2	-4.91	0.16	-30.64	< 0.001
Climate Isolation3	-1.19	0.14	-8.76	< 0.001
Climate Isolation4	0.16	0.13	1.25	0.21
PC1_1	27.78	0.23	120.96	< 0.001
PC1_2	-5.23	0.23	-22.80	< 0.001
PC1_3	-4.90	0.19	-25.19	< 0.001
PC1_4	3.63	0.18	19.66	< 0.001
PC2_1	-12.44	0.25	-49.91	< 0.001
PC2_2	-12.68	0.27	-47.41	< 0.001
PC2_3	-7.45	0.22	-34.40	< 0.001
PC2_4	-3.70	0.19	-19.17	< 0.001

**Predictor's Importance**		**Model Goodness-of-fit**	
**Predictor Importance**	**Proportion of Null Deviance**	*Null Deviance*	120716.00
Climate Area	0.09	*Residual Deviance*	20738.00
Climate Isolation	0.02	*AIC*	25979.00
PC1	0.24	*McFadden's R2*	0.79
PC2	0.05	Predicted R^2	0.84
Joint Contribution within the geography of climate	0.08		
Joint Contribution within climate itself	0.01		
Joint Contribution between the geography of climate and climate itself	0.34		
Total	0.82		

Supplementary Table 22 | Degree-4 Polynomial Regression Outcomes for Reptile Richness in Relation to Geography of Climate and Climate Itself, Assessed Across Various Climate Space Resolutions. This table showcases the impact of geography of climate (climate area and climate isolation) and climate itself (principal components one and two) on bird richness, as determined by a degree-4 polynomial regression. The analysis was carried out using different grids of climate spaces, divided into 20, 30, 40, 50, and 60 equal intervals along the climate axis. The approach of deviance reduction, as outlined in Supplementary Table 1, was applied here to assess the importance of each predictor. The p-values for each variable are computed using two-sided tests.

Reptiles (20 equal intervals)

	Estimate	Std. Error	z value	Pr(>|z|)
Intercept	5.69	0.01	906.15	< 0.001
Climate Area1	6.66	0.07	94.45	< 0.001
Climate Area2	-2.96	0.05	-54.44	< 0.001
Climate Area3	1.60	0.05	33.89	< 0.001
Climate Area4	-0.66	0.04	-16.60	< 0.001
Climate Isolation1	5.59	0.11	49.42	< 0.001
Climate Isolation2	-2.57	0.09	-29.55	< 0.001
Climate Isolation3	-0.72	0.08	-9.00	< 0.001
Climate Isolation4	-0.37	0.06	-6.08	< 0.001
PC1_1	10.59	0.11	96.98	< 0.001
PC1_2	-1.55	0.12	-13.29	< 0.001
PC1_3	-1.94	0.08	-25.40	< 0.001
PC1_4	3.01	0.08	37.01	< 0.001
PC2_1	1.60	0.14	11.74	< 0.001
PC2_2	-5.36	0.12	-44.84	< 0.001
PC2_3	-0.28	0.10	-2.82	< 0.001
PC2_4	-1.37	0.09	-16.05	< 0.001

Predictor's Importance		Model Goodness-of-fit	
**Predictor Importance**	**Proportion of Null Deviance**	*Null Deviance*	105891.00
Climate Area	0.10	*Residual Deviance*	9700.00
Climate Isolation	0.04	*AIC*	10846.00
PC1	0.17	*McFadden's R2*	0.90
PC2	0.03	Predicted R^2	0.94
Joint Contribution within the geography of climate	0.09		

Joint Contribution within climate itself	0.01
Joint Contribution between the geography of climate and climate itself	0.47
Total	0.91

Reptiles (30 equal intervals)

	Estimate	Std. Error	z value	Pr(>|z|)
Intercept	5.44	0.01	1056.23	< 0.001
Climate Area1	8.60	0.07	117.92	< 0.001
Climate Area2	-4.45	0.06	-76.89	< 0.001
Climate Area3	2.56	0.05	54.83	< 0.001
Climate Area4	-1.46	0.04	-35.92	< 0.001
Climate Isolation1	7.99	0.12	67.59	< 0.001
Climate Isolation2	-2.30	0.09	-24.28	< 0.001
Climate Isolation3	-0.47	0.08	-6.07	< 0.001
Climate Isolation4	-0.31	0.07	-4.38	< 0.001
PC1_1	15.82	0.12	131.75	< 0.001
PC1_2	-0.96	0.12	-8.03	< 0.001
PC1_3	-2.28	0.09	-26.60	< 0.001
PC1_4	5.26	0.09	58.20	< 0.001
PC2_1	1.73	0.15	11.91	< 0.001
PC2_2	-7.09	0.14	-50.17	< 0.001
PC2_3	-0.56	0.11	-5.17	< 0.001
PC2_4	-1.12	0.10	-11.56	< 0.001

Predictor's Importance		Model Goodness-of-fit	
**Predictor Importance**	**Proportion of Null Deviance**	*Null Deviance*	148460.00
Climate Area	0.11	*Residual Deviance*	14876.00
Climate Isolation	0.04	*AIC*	16945.00
PC1	0.21	*McFadden's R2*	0.89
PC2	0.02	Predicted R^2	0.92
Joint Contribution within the geography of climate	0.01		
Joint Contribution within climate itself	0.02		
Joint Contribution between the geography of climate and climate itself	0.40		

Total	0.90

Reptiles (40 equal intervals)

	Estimate	Std. Error	z value	Pr(>|z|)
Intercept	5.27	0.00	1200.40	< 0.001
Climate Area1	9.89	0.08	129.11	< 0.001
Climate Area2	-4.52	0.06	-74.26	< 0.001
Climate Area3	2.64	0.05	51.65	< 0.001
Climate Area4	-1.53	0.04	-36.25	< 0.001
Climate Isolation1	8.27	0.11	72.03	< 0.001
Climate Isolation2	-1.82	0.10	-17.71	< 0.001
Climate Isolation3	-1.71	0.09	-19.40	< 0.001
Climate Isolation4	0.41	0.08	5.00	< 0.001
PC1_1	19.51	0.13	150.51	< 0.001
PC1_2	-3.42	0.13	-27.28	< 0.001
PC1_3	-2.16	0.10	-22.43	< 0.001
PC1_4	5.45	0.09	57.77	< 0.001
PC2_1	0.54	0.14	3.70	< 0.001
PC2_2	-8.06	0.15	-55.31	< 0.001
PC2_3	-1.72	0.11	-15.67	< 0.001
PC2_4	-2.06	0.10	-21.10	< 0.001

Predictor's Importance		Model Goodness-of-fit	
**Predictor Importance**	**Proportion of Null Deviance**		
Climate Area	0.10	*Null Deviance*	184517.00
Climate Isolation	0.04	*Residual Deviance*	19744.00
PC1	0.22	*AIC*	22973.00
PC2	0.02	*McFadden's R2*	0.88
Joint Contribution within the geography of climate	0.08	Predicted R^2	0.91
Joint Contribution within climate itself	0.01		
Joint Contribution between the geography of climate and climate itself	0.42		
Total	0.42		

Reptiles (50 equal intervals)

	Estimate	Std. Error	z value	Pr(>|z|)

Intercept	5.14	0.00	1296.97	< 0.001
Climate Area1	11.13	0.08	139.92	< 0.001
Climate Area2	-5.67	0.07	-83.74	< 0.001
Climate Area3	3.28	0.06	57.79	< 0.001
Climate Area4	-1.71	0.05	-35.97	< 0.001
Climate Isolation1	7.38	0.11	65.64	< 0.001
Climate Isolation2	-3.03	0.10	-31.42	< 0.001
Climate Isolation3	-1.22	0.08	-15.04	< 0.001
Climate Isolation4	1.44	0.08	18.87	< 0.001
PC1_1	23.95	0.13	177.82	< 0.001
PC1_2	-4.93	0.12	-39.97	< 0.001
PC1_3	-3.53	0.10	-36.03	< 0.001
PC1_4	6.70	0.09	71.01	< 0.001
PC2_1	-2.67	0.14	-18.51	< 0.001
PC2_2	-8.73	0.15	-58.78	< 0.001
PC2_3	-2.86	0.11	-25.33	< 0.001
PC2_4	-2.65	0.10	-26.12	< 0.001

**Predictor's Importance**		**Model Goodness-of-fit**	
**Predictor Importance**	**Proportion of Null Deviance**	*Null Deviance*	212852.00
Climate Area	0.10	*Residual Deviance*	25316.00
Climate Isolation	0.04	*AIC*	29778.00
PC1	0.27	*McFadden's R2*	0.86
PC2	0.02	Predicted R^2	0.90
Joint Contribution within the geography of climate	0.08		
Joint Contribution within climate itself	0.00		
Joint Contribution between the geography of climate and climate itself	0.37		
Total			

Reptiles (60 equal intervals)

	Estimate	Std. Error	z value	Pr(>|z|)
Intercept	5.02	0.00	1414.05	< 0.001
Climate Area1	12.41	0.08	148.04	< 0.001
Climate Area2	-5.84	0.07	-82.36	< 0.001

Climate Area3	3.43	0.06	54.55	< 0.001
Climate Area4	-1.96	0.05	-37.67	< 0.001
Climate Isolation1	7.14	0.11	63.44	< 0.001
Climate Isolation2	-1.69	0.10	-17.28	< 0.001
Climate Isolation3	-2.25	0.08	-28.39	< 0.001
Climate Isolation4	0.66	0.08	8.62	< 0.001
PC1_1	27.80	0.15	190.52	< 0.001
PC1_2	-6.72	0.13	-50.73	< 0.001
PC1_3	-3.62	0.11	-32.78	< 0.001
PC1_4	7.62	0.10	73.21	< 0.001
PC2_1	-3.96	0.16	-25.29	< 0.001
PC2_2	-10.92	0.17	-65.89	< 0.001
PC2_3	-3.70	0.13	-28.81	< 0.001
PC2_4	-3.50	0.11	-31.13	< 0.001

**Predictor's Importance**		**Model Goodness-of-fit**	
**Predictor Importance**	**Proportion of Null Deviance**	*Null Deviance*	241519.00
Climate Area	0.10	*Residual Deviance*	30888.00
Climate Isolation	0.02	*AIC*	36847.00
PC1	0.29	*McFadden's R2*	0.85
PC2	0.03	Predicted R^2	0.89
Joint Contribution within the geography of climate	0.08		
Joint Contribution within climate itself	0.01		
Joint Contribution between the geography of climate and climate itself	0.34		
Total	0.87		
