# Supplementary material for: The geography of climate and the global patterns of species diversity
Source: Nature. 2023 Sep 27;622(7983):537–44. doi: 10.1038/s41586-023-06577-5 (PMC10584679; doi:10.1038/s41586-023-06577-5)
Supplement: Supplementary file 2 — Reporting Summary [file 41586_2023_6577_MOESM2_ESM.pdf]

nature portfolio

Corresponding author(s): Marco Túlio Pacheco Coelho

Last updated by author(s): Aug 17, 2023

Reporting Summary

Nature Portfolio wishes to improve the reproducibility of the work that we publish. This form provides structure for consistency and transparency in reporting. For further information on Nature Portfolio policies, see our Editorial Policies and the Editorial Policy Checklist.

Statistics

For all statistical analyses, confirm that the following items are present in the figure legend, table legend, main text, or Methods section.

n/a	Confirmed	
☐	☒	The exact sample size (*n*) for each experimental group/condition, given as a discrete number and unit of measurement
☒	☐	A statement on whether measurements were taken from distinct samples or whether the same sample was measured repeatedly
☐	☒	The statistical test(s) used AND whether they are one- or two-sided<br>*Only common tests should be described solely by name; describe more complex techniques in the Methods section.*
☐	☒	A description of all covariates tested
☐	☒	A description of any assumptions or corrections, such as tests of normality and adjustment for multiple comparisons
☐	☒	A full description of the statistical parameters including central tendency (e.g. means) or other basic estimates (e.g. regression coefficient) AND variation (e.g. standard deviation) or associated estimates of uncertainty (e.g. confidence intervals)
☐	☒	For null hypothesis testing, the test statistic (e.g. *F*, *t*, *r*) with confidence intervals, effect sizes, degrees of freedom and *P* value noted<br>*Give P values as exact values whenever suitable.*
☒	☐	For Bayesian analysis, information on the choice of priors and Markov chain Monte Carlo settings
☒	☐	For hierarchical and complex designs, identification of the appropriate level for tests and full reporting of outcomes
☐	☒	Estimates of effect sizes (e.g. Cohen's *d*, Pearson's *r*), indicating how they were calculated

Our web collection on statistics for biologists contains articles on many of the points above.

Software and code

Policy information about availability of computer code

Data collection	In the course of this study, we did not utilize any specialized software to collect our data.
Data analysis	Data analysis was performed in R version 4.1.2 (2021-11-01) on platform aarch64-apple-darwin20. The code used for statistical analysis is available as supporting material.

For manuscripts utilizing custom algorithms or software that are central to the research but not yet described in published literature, software must be made available to editors and reviewers. We strongly encourage code deposition in a community repository (e.g. GitHub). See the Nature Portfolio guidelines for submitting code & software for further information.

Data

Policy information about availability of data

All manuscripts must include a data availability statement. This statement should provide the following information, where applicable:

- Accession codes, unique identifiers, or web links for publicly available datasets
- A description of any restrictions on data availability
- For clinical datasets or third party data, please ensure that the statement adheres to our policy

Global climate and biological data used in our study are open source. All climate data is available at CHELSA (https://chelsa-climate.org/bioclim/) and CGIAR (https://cgiarcsi.community). Vector range maps of all amphibians and mammals are available at IUCN (iucn.org). Bird and squamate range maps are available respectively on Birdlife (Version 2020.1, http://datazone.birdlife.org/species/) and Roll, U. et al. (https://doi.org/10.1038/s41559-017-0332-2).

Research involving human participants, their data, or biological material

Policy information about studies with human participants or human data. See also policy information about sex, gender (identity/presentation), and sexual orientation and race, ethnicity and racism.

Reporting on sex and gender	Not applicable.
Reporting on race, ethnicity, or other socially relevant groupings	Not applicable.
Population characteristics	Not applicable.
Recruitment	Not applicable.
Ethics oversight	Not applicable.

Note that full information on the approval of the study protocol must also be provided in the manuscript.

Field-specific reporting

Please select the one below that is the best fit for your research. If you are not sure, read the appropriate sections before making your selection.

☐ Life sciences ☐ Behavioural & social sciences ☒ Ecological, evolutionary & environmental sciences

For a reference copy of the document with all sections, see nature.com/documents/nr-reporting-summary-flat.pdf

Ecological, evolutionary & environmental sciences study design

All studies must disclose on these points even when the disclosure is negative.

Study description	In this study, we conducted a global assessment to analyze the influence of climate and geographical characteristics - including fragmentation and size - on the richness and composition of tetrapods. We developed a two-dimensional orthogonal climate space, defined by twelve global scale climate variables, using multiple resolutions from fine to coarse, and considering different definitions of climate space. This enabled us to measure species diversity and compositional dissimilarities for each uniquely classified climate. Each climate was defined by its size, degree of fragmentation and dispersion across the globe, and intrinsic climatic characteristics. These parameters served as predictor variables, which we analyzed in relation to species richness and composition. Our analytical approach involved both linear and non-linear multivariate models, with the non-linear models being tested using different implementation methods. This comprehensive approach allowed us to account for a variety of climatic variables and better understand their effects on tetrapod richness and composition.
Research sample	In our study, we first established the presence and absence of various tetrapod groups, specifically amphibians, reptiles, birds, and mammals, across more than 30,000 species, within each classified climate. The climate classification was global, implying that our study also spans a global scale. To execute this classification, we utilized twelve distinct climatic variables that represent the current climate. This approach allowed us to accurately determine the distribution and prevalence of the studied species across different climate classifications worldwide.
Sampling strategy	Given the global scale of our research, our approach was designed to encompass a comprehensive scope, ensuring the inclusion of all pertinent data in order to effectively capture the richness and composition of tetrapods across various climatic classifications worldwide. Thus, we utilized all relevant data available to us without predetermining sample size through statistical methods.
Data collection	All data for this study were derived from existing datasets, and were collected online by the authors themselves.
Timing and spatial scale	The spatial scale of this study is global, with both biodiversity and climate data representing current conditions. As such, our findings provide a snapshot of the present state of tetrapod richness and composition in relation to various climatic classifications worldwide.
Data exclusions	No data were excluded from the analyses.
Reproducibility	This research does not fall into the category of experimental studies; rather, it is an observational study. Consequently, traditional experimental reproducibility is not applicable.
Randomization	This research does not fall into the category of experimental studies; rather, it is an observational study. Consequently, traditional experimental randomization is not applicable.
Blinding	This research does not fall into the category of experimental studies; rather, it is an observational study. Consequently, blinding is not applicable.

Did the study involve field work? ☐ Yes ☒ No

Reporting for specific materials, systems and methods

We require information from authors about some types of materials, experimental systems and methods used in many studies. Here, indicate whether each material, system or method listed is relevant to your study. If you are not sure if a list item applies to your research, read the appropriate section before selecting a response.

Materials & experimental systems

n/a	Involved in the study	
☒	☐	Antibodies
☒	☐	Eukaryotic cell lines
☒	☐	Palaeontology and archaeology
☒	☐	Animals and other organisms
☒	☐	Clinical data
☒	☐	Dual use research of concern
☒	☐	Plants

Methods

n/a	Involved in the study	
☒	☐	ChIP-seq
☒	☐	Flow cytometry
☒	☐	MRI-based neuroimaging
